# Ruthenium polypyridine complexes with triphenylamine groups as antibacterial agents against *Staphylococcus aureus* with membrane-disruptive mechanism

**DOI:** 10.3389/fchem.2022.1035741

**Published:** 2022-10-10

**Authors:** Li Jiang, Yuanyuan Ma, Yanshi Xiong, Yanhui Tan, Xuemin Duan, Xiangwen Liao, Jintao Wang

**Affiliations:** ^1^ Jiangxi Provincial Key Laboratory of Drug Design and Evaluation, School of Pharmacy, Jiangxi Science & Technology Normal University, Nanchang, China; ^2^ State Key Laboratory for Chemistry and Molecular Engineering of Medicinal Resources, School of Chemistry and Pharmaceutical Sciences, Guangxi Normal University, Guilin, China

**Keywords:** Ru(II) complexes, antimicrobial properties, antibiofilm activity, synergistic effect, membrane-disruptive mechanism

## Abstract

Due to the emergence and wide spread of methicillin-resistant *Staphylococcus aureus*, the treatment of this kind of infection becomes more and more difficult. To solve the problem of drug resistance, it is urgent to develop new antibiotics to avoid the most serious situation of no drug available. Three new Ru complexes [Ru (dmob)_2_PMA] (PF6)_2_ (**Ru-1**) [Ru (bpy)_2_PMA] (PF6)_2_ (**Ru-2**) and [Ru (dmb)_2_PMA] (PF6)_2_ (**Ru-3**) (dmob = 4,4′-dimethoxy-2,2′-bipyridine, bpy = 2,2′-bipyridine, dmb = 4,4′-dimethyl-2,2′-bipyridine and PMA = N-(4-(1H-imidazo [4,5-f] [1,10] phenanthrolin-2-yl) -4-methyl-N-(p-tolyl) aniline) were synthesized and characterized by 1H NMR, 13C NMR and HRMS. The detailed molecular structure of **Ru-3** was determined by single crystal X-ray diffraction. Their antibacterial activities against *Staphylococcus aureus* (*Staphylococcus aureus*) were obvious and **Ru-3** showed the best antibacterial effect with the minimum inhibitory concentration value of 4 μg ml^−1^. Therefore, further study on its biological activity showed that **Ru-3** can effectively inhibit the formation of biofilm and destroy cell membrane. *In vitro* hemolysis test showed that **Ru-3** has almost negligible cytotoxicity to mammalian red blood cells. In the toxicity test of wax moth insect model, **Ru-3** exhibited low toxicity *in vivo*. These results, combined with histopathological studies, strongly suggest that **Ru-3** was almost non-toxic. In addition, the synergistic effect of **Ru-3** with common antibiotics such as ampicillin, chloramphenicol, tetracycline, kanamycin and gentamicin on *Staphylococcus aureus* was detected by chessboard method. Finally, *in vivo* results revealed that **Ru-3** could obviously promote the wound healing of *Staphylococcus aureus* infected mice.

## Introduction

Since the discovery of penicillin, antibiotics have saved countless lives, prevented fatal infections and made great contributions to the Figureht against human infectious diseases. However, the extensive and evolving pathogenic behavior of bacteria and the abuse and misuse of antibiotics lead to a sharply increase in bacterial drug resistance, which poses a serious threat to public health (Aslam et al., 2018; Tacconelli et al., 2018; Richter and Hergenrother, 2019; De Oliveira et al., 2020; Laxminarayan et al., 2020; Song et al., 2020). It is predicted that drug-resistant infection may cause 10 million deaths every year by 2050 (Piddock, 2016; Abouelhassan et al., 2019). *Staphylococcus aureus* is one of the most common causes of hospital and community-acquired infection, which is closely related to pneumonia, endocarditis, osteomyelitis, arthritis and sepsis (Hussain et al., 2018). Due to the emergence and wide spread of methicillin-resistant *Staphylococcus aureus*, the treatment of this kind of infection becomes more and more difficult. To solve the problem of drug resistance, it is urgent to develop new antibiotics to avoid the most serious situation of no drug available.

It is widely accepted that some transition metal complexes have more advantages than traditional organic molecular drugs, such as easy structural modification, rich photophysical and electrochemical properties (Gitlin and Lill, 2012; Howerton et al., 2012; Knoll and Turro, 2015) Among them, polypyridine ruthenium (II) complexes have a wide range of potential properties, such as DNA binding agents, antibacterial agents and anticancer agents (Li et al., 2015; Mesquita et al., 2018; Moumita et al., 2021). So far, a few ruthenium (II) complexes have been reported as antibacterial agents (Patra, et al., 2012; Gorle et al., 2014; Nyawade, et al., 2015; Srivastava, et al., 2019; Lei, et al., 2020; Singh and Barman, 2021; Sun et al., 2021; Varney et al., 2021) and generally ruthenium (II) complexes were more active than theirs’ coordinative ligands (Carlsen et al., 1981; Weber et al., 2016). Moreover, some of that reported Ru(II) polypyridine complexes with different functional groups simultaneously exhibited interesting synergy effects between existing common antibiotics, which were potential adjuvants to enhance the effect of existing antibiotics on *Staphylococcus aureus* (Liao et al., 2020; Zhang et al., 2022).

**FIGURE 1:**
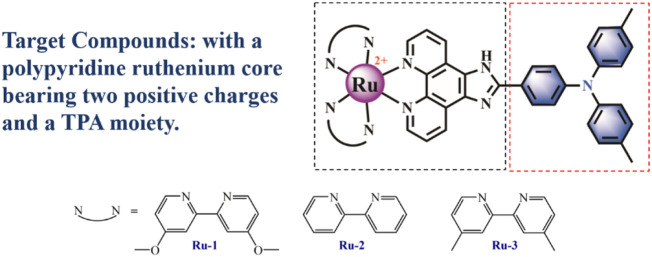
Structures and design stratege for target Ru(II) complexes.

Recently, Tang and co-workers explored a multifunctional TPA derivative, which showed good selective sterilization effect and targeted Gram-positive bacteria. In addition, that compound could destroy the cell membrane of *Staphylococcus aureus* under white light irradiation and had important anti-infective effect *in vivo* (Kang et al., 2019; Li et al., 2022; Liu et al., 2022). Inspired by the above research, herein, three new Ru(II) complexes (Figure 1) with TPA derivatives were designed and synthesized [Ru (domb)_2_PMA] (PF6)_2_ (**Ru-1**) [Ru (bpy)_2_PMA] (PF6)_2_ (**Ru-2**) and [Ru (dmb)_2_PMA] (PF6)_2_ (**Ru-3**). Their antibacterial activity against *Staphylococcus aureus* was evaluated. In addition, considering the toxicity and drug resistance of **Ru-3** with the best activity, its behaviors concerning antibacterial adjuvant, bacterial biofilm destruction, antibacterial mechanism, antibacterial activity *in vivo* were further explored.

## Materials and methods

All reagents and materials were purchased from commercial suppliers used as received without further purification. 4-di-p-tolylamino benzaldehyde and 1,10-phenanthroline-hydrate were purchased from Energy-chemical. Rabbit blood was purchased from Maojie Microbes. 3, 3′- dipropylthiadicarbocyanine iodide [DiSC_3_(5)] was purchased from Macklin. 4′,6-diamidino-2-phenylindole (DAPI) was purchased from Solarbio and propidium Iodide (PI) was obtained from 3 A Materials. All antibiotics and crystal violet were obtained from Sangon. Tryptic soy broth (TSB) was purchased from Hapebio. Agar powder was purchased from Chembase. *Staphylococcus aureus* strain was obtained from China Center of Industrial Culture Collection (CICC). The starting materials [Ru(dmob)_2_Cl_2_] [Ru(bpy)_2_Cl_2_] and [Ru(dmb)_2_Cl_2_] were synthesized according to the literature (Sullivan et al., 1978; Collin and Sauvage, 1986; Castellano et al., 1998; Tran et al., 2013).

Nuclear magnetic spectra were recorded on a Bruker AVANCE 400 spectrometer under ambient conditions. High resolution mass spectrometric analysis was carried out on a Waters Xevo G2-XS Q-TOF instrument. A Shimadzu UV-2550 UV-vis spectrophotometer was used for UV scanning. A biochemical incubator and constant temperature culture shaker were purchased from Yiheng Scientific Instruments. Enzyme-labeled instrument was obtained from BioTek Instruments and a fluorescent cell imager was purchased from BIO-RAD.

### Synthesis and characterization PMA

The ligand N-(4-(1H-imi-dazo[4,5-f][1,10]phenanthrolin-2-yl(phenyl)-4-methyl-N-(p-tolyl)aniline was synthesized according to the literature (Zhang et al., 2011; Peng et al., 2021). ^1^H NMR (400 MHz, CDCl_3_) *δ* 8.82 (s, 4H), 8.08 (d, J = 8.1 Hz, 2H), 7.39 (s, 2H), 7.05–6.84 (m, 10H), 2.25 (s, 6H). HRMS (acetonitrile) m/z: calcd 492.2144 for C_33_H_25_N_5_, found 492.2185 for [PAM + H]^+^.

### Synthesis and characterization Ru-1

([Ru(dmob)_2_PMA] (PF_6_)_2_). A mixture of [Ru(dmob)_2_Cl_2_] (55.2 mg, 0.1 mmol) and PMA (49.8 mg, 0.1 mmol) in ethylene glycol (10 ml) was heated at 150°C under argon for 8 h to give a clear red solution. After cooling to room temperature, a red precipitate was obtained after 1 mmol KPF_6_ aqueous solution (50 ml) was added. The crude product was purified by column chromatography on neutral alumina with a CH_3_CN/Xylene mixture as the eluent to obtain a red powder. Yield: 56.5 mg (55.1%). ^1^H NMR (400 MHz, DMSO-*d_6_*) *d* 14.12 (s, 1H), 9.07 (d, J = 31.6 Hz, 2H), 8.49 (d, J = 18.0 Hz, 4H), 8.14 (d, J = 5.6 Hz, 4H), 7.90 (s, 2H), 7.64 (d, J = 6.4 Hz, 2H), 7.28 (s, 2H), 7.20 (d, J = 8.2 Hz, 6H), 7.06 (t, J = 10.2 Hz, 6H), 6.92 (s, 2H), 4.03 (s, 6H), 3.93 (s, 6H), 2.31 (s, 6H). ^13^C NMR (101 MHz, DMSO-*d_6_*) *d* 166.46, 166.30, 157.79, 151.85, 151.64, 149.64, 149.50, 143.89, 133.42, 130.18, 129.41, 127.67, 125.85, 125.24, 121.30, 120.07, 114.04, 113.86, 111.16, 56.66, 56.56, 20.32. HRMS (acetonitrile) m/z: calcd 512.6476 for [C_57_H_49_N_9_O_4_Ru]^2+^, found 512.6492 for [M-2PF_6_]^2+^.

### Synthesis and characterization Ru-2

([Ru(bpy)_2_PMA] (PF_6_)_2_). This complex was synthesized in an identical manner as described for complex **Ru-1** using a mixture of [Ru(bpy)_2_Cl_2_] (147.4 mg, 0.3 mmol) and PMA (145.3 mg, 0.3 mmol). Yield: 280.8 mg (78.3%). ^1^H NMR (400 MHz, DMSO-*d_6_*) *d* 14.16 (s, 1H), 9.05 (d, J = 8.2 Hz, 2H), 8.77 (d, J = 8.2 Hz, 4H), 8.39 (s, 4H), 8.20–7.94 (m, 8H), 7.84–7.70 (m, 6H), 7.20 (d, J = 7.9 Hz, 4H), 7.04 (d, J = 8.0 Hz, 6H), 2.31 (s, 6H). ^13^H NMR (101 MHz, DMSO-*d_6_*) *d* 152.78, 152.68, 152.50, 149.99, 147.16, 147.08, 145.12, 143.87, 136.68, 133.41, 130.34, 130.18, 127.92, 127.64, 126.18, 125.89, 125.24, 120.03, 20.33. HRMS (acetonitrile) m/z: calcd 452.6264 for [C_53_H_41_N_9_Ru]^2+^, found 452.6285 for [M-2PF_6_]^2+^.

### Synthesis and characterization Ru-3

([Ru(dmb)_2_PMA] (PF_6_)_2_). This complex was synthesized in an identical manner as described for complex **Ru-1**, with [Ru (dmb)_2_Cl_2_] in place of [Ru(domb)_2_Cl_2_]. Yield: 121.3 mg (55.6%). ^1^H NMR (400 MHz, DMSO-*d_6_*) *δ* 14.09 (s, 1H), 9.04 (s, 2H), 8.71 (d, J = 18.3 Hz, 4H), 8.13 (s, 2H), 8.03 (s, 2H), 7.89 (s, 4H), 7.66 (s, 2H), 7.51 (s, 2H), 7.40 (s, 4H), 7.19 (s, 4H), 7.04 (s, 4H), 2.56 (s, 6H), 2.46 (s, 6H), 2.31 (s, 6H). ^13^C NMR (101 MHz, DMSO-*d_6_*) *δ* 156.22, 156.06, 150.26, 149.40, 149.20, 144.80, 143.93, 133.30, 130.14, 129.82, 128.34, 128.21, 127.61, 125.84, 125.14, 124.80, 120.18, 20.59, 20.50, 20.30. HRMS (acetonitrile) m/z: calcd 480.6577 for [C_57_H_49_N_9_Ru]^2+^, found 480.6597 for [M-2PF_6_]^2+^.

### Single crystal X-ray data

Collection and Structure Refinement. The monocrystal data of the **Ru-3** (0.11 × 0.06 × 0.04 mm) were collected using an Agilent Gemini EOS diffractometer with graphite-monochromated with Mo-Kα radiation (*λ* = 0.71073) at 170 K. An empiric absorption correction was applied. All the non-hydrogen atoms were refined anisotropically and the hydrogen atoms of organic molecule were refined in calculated positions, assigned isotropic thermal parameters, and allowed to ride their parent atoms. All calculations were performed using the SHELX2014 program package (Sheldrick, 2015). Crystallographic data (excluding structure factors) for the structure of **Ru-3** in this paper have been deposited with the Cambridge Crystallographic Data Centre with the reference numbers 2165862.

### Antibacterial activity

Antibacterial activity was evaluated by measuring MIC (minimum inhibitory concentration) and MBC (minimum bactericidal concentration) values. The MIC value was measured by the microdilution method using TSB broth with 96 well plates (Carlsen et al., 1981) and the MBC value was determined by LB plates. In brief, the overnight cultured bacteria were 1: 1000 diluted with fresh medium to get a bacterial suspension. After incubation at 37°C for 20 h, the growth of bacteria is monitored by observing the turbidity of the culture. The bacterial solution was diluted as above method and the complexes were incubated with bacterial solution for 2 h. Then 100 μL bacterial solution was taken for plate coating. The *Staphylococcus aureus* growth inhibition trend in the presence of complexes was obtained. The MBC values of complexes were determined by LB plates after culturing in the same method for 24 h. All experiments were controlled with sterile water and repeated in parallel at least three times.

### Hemolytic activity

Obtain red blood cells from fresh sterile rabbit blood and rabbit blood was washed three times with PBS. Ru(II) complexes in 950 µL phosphoric acid buffer (PBS) of different concentrations and 50 μL red blood cells were added into a 1.5 ml sample tube, incubated at 37°C for 30 min. The negative control was red blood cell suspension containing only PBS, and the positive control was PBS containing 0.1% (V/V) Triton X-100. After incubating the mixture was centrifugated (2000 rpm for 2 min) and the supernatant (200 μL) was transfered to another 96 well plate. Finally, the hemolysis rate was calculated by measuring the absorbance at 540 nm.

### Effect of ruthenium complex on the growth of *Staphylococcus aureus*

The effect of ruthenium complexes on the growth curve of *Staphylococcus aureus* was determined. Briefly, overnight cultured *Staphylococcus aureus* was diluted 1:1000 with fresh broth medium. Then the bacterial culture and ruthenium complexes were placed in a 24 well Petri dish and shaken at 37°C. After, the OD_600_ of bacteria was measured every 30 min for 20 h. Data analysis was carried out with Graphad Prism.

### Determination of distribution coefficient

The partition coefficients of all complexes were determined by standard shake flask method in 1-octanol and buffer liquid system (Yang et al., 2021). In brief, the octanol/water partition coefficient is obtained by the incubation of 2 ml of 25 μg ml^−1^ ruthenium complex 1-octanol and 2 ml PBS samples. After shaking the solution for 6 h, the samples were stood for 2 h. The absorbance of octanol complex at 282 nm before and after oscillation was measured. The absorbance of 1-octanol before oscillation minus the absorbance of 1-octanol after oscillation is the absorbance of the complex in water. Each experiment was repeated three times. The partition coefficient is reported as the number of octanol divided by the number of water.

### Effect of Ru-3 on biofilm formation

24 well plate was used for biofilm determination. The overnight cultured *Staphylococcus aureus* strain was diluted 1000 times with fresh TSB medium. Then 2 ml of that diluted bacterial solution was mixed with 500 µL **Ru-3** of different concentrations in a 24 well plate. After incubation at 37°C for 48 h, the bacterial suspension was removed and the plate was washed three times with PBS. The adherent bacteria were dried overnight at 37°C and then dyed through 0.1% crystal violet solution. After 2 min, taking out the crystal violet solution and wash the plate with PBS again. And then, adding 1 ml acetic acid and 1 ml water, the formation of biofilm can be determined by monitoring the absorbance at 595 nm.

### Ru-3 killing bacteria in biofilm

To establish bacterial biofilm, the overnight cultured *Staphylococcus aureus* strain was diluted 1000 times with fresh TSB medium, then the bacterial suspension was transferred to 96 well plate and cultured for 24 h. Subsequently, the supernatant was removed and the formed biofilm was washed 3 times with PBS solution. 200 μL solution containing **Ru-3** of different concentrations (512, 256, 128, 64, 32, 16, 8, 4 μg ml^−1^) were added and further incubated at 37°C for 24 h. Biofilm without **Ru-3** were used as positive controls. Then the supernatant was discarded and the residual biofilm was cleaned using 200 μL PBS 3 times. Next, fresh medium was added to culture for 24 h (Yang et al., 2021). The solution after culture was diluted 1000 times and 100 μL was taken out for plate coating and counting.

### Study on drug resistance of bacterial

After *Staphylococcus aureus* was cultured for 5 h, the bacteria were diluted 1000 times with fresh TSB and the MIC of **Ru-3** was measured. The bacterial solution grown at the sub inhibitory concentration of compound **Ru-3** was inoculated into fresh TSB medium for 5 h, then the MIC was measured, and the above procedure was repeated for 20 generations. Ampicillin was used as the control.

### Checkerboard assay

Firstly, the MIC of all selected antibiotics was determined by the above method. *Staphylococcus aureus* was cultured overnight and diluted 1000 times with fresh TSB. Then 200 μL the diluted bacterial suspension, 25 μL **Ru-3** of gradient concentrations and 25 μL antibiotics of gradient concentrations were mixed in 96 well plates and further cultured at 37°C for 20 h. MIC values of single drug and the best combination effect (combination of MICA and MICB) were measured. Graphpad prism software was used to draw the checkerboard map and isoline map.

### Secrete toxins

Firstly, *S. aureus* was cultured overnight and diluted 1000 times with fresh TSB, then a mixture of **Ru-3** of 1 μg ml^−1^ or 2 μg ml^−1^ and *Staphylococcus aureus* solution was cultured in a shaking Table at 37°C for 18 h. After culturing, centrifuging (5000 rpm, 2 min) *Staphylococcus aureus* solution. And rabbit blood cells were prepared with PBS buffer (washing rabbit blood three times). Blood cells were collected by centrifugation (2000 rpm, 2 min). Secondly, a mixture containing 1 ml PBS buffer, 150 μL supernatant and 25 μL blood cells was cultured at 37°C for 30 min. Then cultured supernatant was obtained by centrifugation (2000 rpm, room temperature, 2 min). Finally, the optical density of the supernatant was measured at 540 nm.

### Nucleic acid leakage

To verify the membrane damage, the loss of 260 nm absorbing material was carried out (Yu et al., 2021). Briefly, overnight cultured bacteria were diluted 1:1000 in fresh TSB and shaken at 37°C for about 5 h until the exponential stage was reached. The supernatant was removed by centrifugation, then the bacteria were resuspended to OD_600_ = 1 with PBS, which was further treated with **Ru-3** or polymyxin B and centrifuged after 2 h to precipitate bacterial cells. Subsequently, the loss of 260 nm absorbing material, including the release of DNA and RNA in the filtrate, was measured at 260 nm.

### Effect of Ru-3 on bacterial cell morphology

Scanning electron microscope (SEM) is an important method to observe cell morphology. Briefly, *S. aureus* was cultured in TSB medium to exponential phase, which were collected and washed with PBS three times by centrifugation. After, the bacterial precipitate was diluted to OD_600_ = 0.3 with PBS. Using sterile water as the blank control, **Ru-3** (4 μg ml^−1^) was added to the bacterial suspension solution for 2 h. After Incubating, the bacteria were fixed with 2.5% glutaraldehyde at 4°C overnight. Pour out the fixed solution and rinse the sample three times with 0.1 M PBS for 15 min each time. The samples were fixed with 1% osmic acid solution for 1–2 h, then the osmic acid was carefully taken out, and the samples were washed three times with 0.1 M PBS for 15 min each time. The samples were then dehydrated by a series of graded concentrations of ethanol (30%, 50%, 70%, 80%, 90% and 95%). The samples were further treated with a mixture of ethanol and isoamyl acetate (V/V = 1/1) for 30 min, then treated with pure isoamyl acetate for overnight. Finally, the treated samples were subjected to critical drying and observed by SEM.

### DiSC_3_(5) and DAPI/PI fluorescence staining

Firstly, *S. aureus* was cultured in TSB medium to exponential phase. Exponential growth bacteria were collected by centrifugation at 5000 rpm for 2 min, then washed with PBS and diluted to OD_600_ value of 0.3. Secondly, **Ru-3** (4 μg ml^−1^) was added to the bacterial suspension solution and incubated at 37°C for 2 h, then the supernatant was removed by centrifuging. Thirdly, the bacterial was washed with PBS for three times and suspendedin 500 μL PBS. Subsequently, adding 20 μL DiSC_3_(5) (30 μM), incubating for 1 h under dark conditions. For DAPI and PI, adding 20 μL DAPI (10 μg ml^−1^), incubating for 15 min under dark conditions. Next, adding 20 μL PI (15 μg ml^−1^) in the same tube, and incubating for 15 min under dark conditions. After that, centrifuge, removing the supernatant. Eventually, suspending with 500 uL PBS and 20 μL samples were transfered on glass slides and observed under fluorescent cell imager.

### Study on membrane permeability

O-nitrobenzene-β-d-galactopyranoside (ONPG) was the substrate of intracellular *ß*-galactosidase, that was used to determine the permeability of *Staphylococcus aureus* cell membrane. *Staphylococcus aureus* was cultured to logarithmic phase, then washed and cultured in M9 lactose medium, in which lactose was used as the only carbon source of a single colony and stayed overnight at 37°C. After washing three times with PBS, the culture was diluted to OD_600_ = 0.3 (PBS). Subsequently, **Ru-3** (4 μg ml^−1^) or vancomycin (2 μg ml^−1^, as positive control) was added to the bacterial suspension solution and then each tube also contains 1.5 mM ONPG, which was further shake at 37°C. The hydrolysis of ONPG to o-nitrophenol over time was monitored by UV every 15 min at 415 nm. A similar procedure was used for untreated cells as control (Xuan et al., 2021).

### *In vivo* antibacterial activity test

*S. aureus* was cultured in TSB medium to exponential phase. Bacterial precipitates were collected and washed with PBS three times by centrifugation, which was diluted to OD_600_ = 1 (1.02 × 10^8^ CFU/ml) with sterile normal saline. The day before the infection, the hair on the back of the mice was shaved off. Then depilatory cream (Veet^®^) was used to remove the remaining hair. Subsequently, 100 μL *S. aureus* was injected in subcutaneous and the abscess formed after 12 h later. All mice infected with *S. aureus* were divided into two groups (n = 5 in each group), including control group and treatment group, **Ru-3** (0.05 mg ml^−1^), which was fully mixed in sterile cream. Afterwards, mouse abscesses were treated with creams containing **Ru-3** 4 times a day. After 10 days, the experiment was ended. The study was conducted in strict accordance with NIH guidelines for the care and use of laboratory animals (NIH Publication No. 85–23, revised in 1985), and was reviewed and approved by the institutional animal care and use Committee of Guangxi Normal University (Guilin, China).

### Acute skin irritation test

Female BALB/c mice were randomly divided into three groups, control group, **Ru-3** (0.05 mg ml^−1^) group and **Ru-3** (0.1 mg ml^−1^). The day before the experiment, the hair on the back of female mice was removed. The compound and distilled water control were gently attached to the shaving site (about 2 cm^2^) once a day for 3 days. On the fourth day, the mice were killed by cervical dislocation. The skin tissue at the site of infections were taken out and fixed in 4% paraformaldehyde at 4°C for 1 day, then embedded in paraffin. Serial sections were prepared for H&E analysis.

## Results and discussion

Synthesis and Characterization. All ligands and complexes were prepared according to the procedure shown in (Supplementary Figure S1) and characterized by 1H NMR spectrum, 13C NMR spectrum, HPLC, UV-Vis analysis and HR-MS spectrum. In the case of three ruthenium complexes (Supplementary Figure S2), the UV-Vis spectral data showed that there were strong bands at 264–287 nm, which may be attributed to the *p*–π* transition, while the relatively weak band in the range of 369–376 nm may correspond to the charge transfer transition from metal to ligand. There cationic complexes were isolated with hexafluorophosphate as the counteranion, making them easy to purify and less moisture-sensitive. Stock solutions (5 mg/ml) of all complexes were prepared in DMSO, which were further diluted using buffer or cell culture medium until working concentrations were achieved. As the complexes need to be stable in the biological environment, the stability of all ruthenium (II) complexes was determined by UV spectra. Three ruthenium (II) complexes dissolved in DMSO was diluted by acetonitrile or H_2_O. There was no significant change in the spectral pattern from 0 to 24 h, as shown in Supplementary Figure S17, which suggests the stability of the complexes in solvent and indicates they can be used for antibacterial activities.

### Crystal structures

**FIGURE 2:**
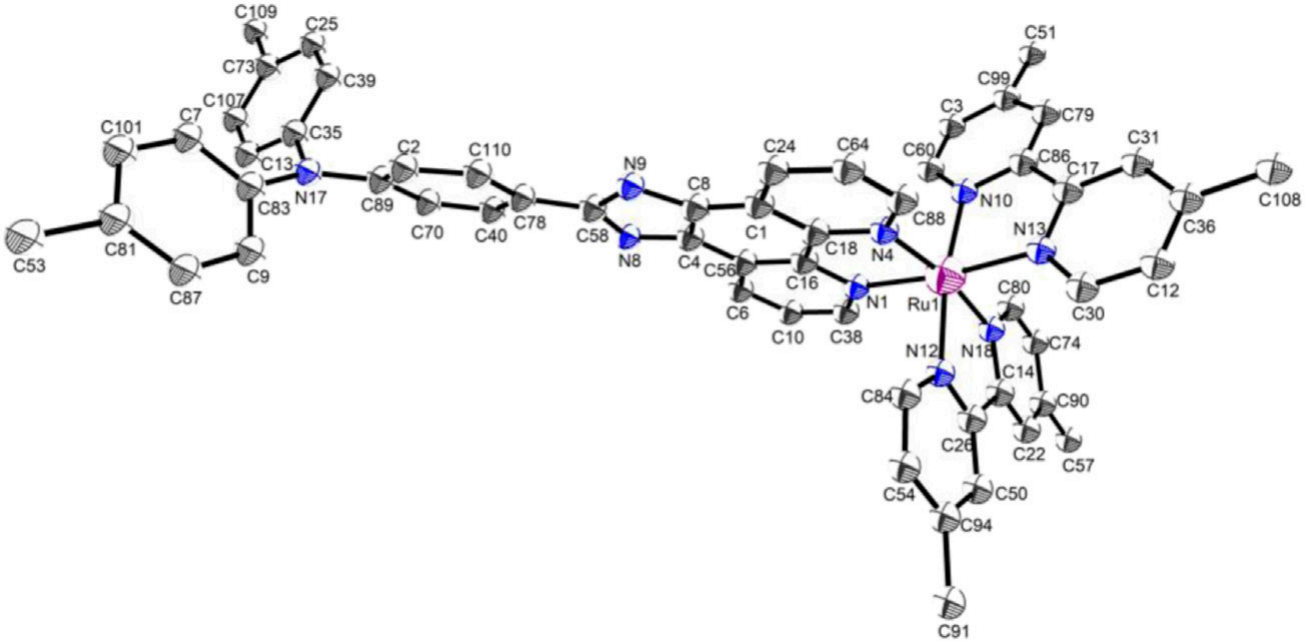
Thermal ellipsoid plot of Ru-3 in ORTEP view.

Red crystals were obtained by volatilization from an acetonitrile and water mixture. The **Ru-3** was characterized by single-crystal X-ray crystallography. **Ru-3** crystallizes in the C2 monoclinic space group. Crystallographic data and structural parameters are listed in Table 1. Information concerning X-ray data collection and crystal structure refinement is summarized in Supplementary Table S1, with the selected bond lengths and bond angles listed in Suplementary Table S2. An ORTEP perspective view of the structure is shown in Figure 2, that proved the synthetic complex with the expected structure, and the obtained bond lengths and bond angles are similar to other reported related ruthenium polypyridine complexes (Liu et al., 2001; Singha et al., 2017). Nevertheless, the most interesting feature of the crystal structure is the mode of π-π electron interactions between pyridine rings of adjacent molecules, and the dihedral angle between ring 1 (N8–N9) and ring 2 (C46–C48) is only 14.28°, showing they are nearly parallel (Supplementary Figure S3). The distance between ring one and ring two is 3.710 Å, suggesting that weak intermolecular *p*-π stacking interactions are involved in stabilizing the monomer structure.

**Table 1:** *In vitro* Antimicrobial Activities Against *S. aureus* and Hemolytic Activities of compounds.

Compounds	MIC µg/mL	MBC µg/mL	% Hemolysis at 256 μg/ml	LgP
Ru-1	16	128	8	1.14
Ru-2	8	16	16	0.97
Ru-3	4	32	4	1.79
PMA	>256	—	—	—
RuCl3∙3H2O	>256	—	—	—
Vancomycin	2	—	—	—

### The antibacterial activity studies

The minimum inhibitory concentration (MIC) and the minimum bactericidal concentration (MBC) of three complexes against *S. aureus* were determined. As shown in Table 1, all designed TPA modified complexes showed interesting antibacterial activity, among which **Ru-3** exhibited the best antibacterial activity (MIC = 4 μg ml^−1^, MBC = 32 μg ml^−1^). Meanwhile, the MBC values of **Ru-1** and **Ru-2** are 16 μg ml^−1^ and 128 μg ml^−1^. On the other hand, free TPA ligands and RuCl_3_∙3H_2_O showed no antibacterial activity (>256 μg ml^−1^). Therefore, the antibacterial results showed that the combination of the ligands and Ru was essential for complexes’ antibacterial activities. In addition, the % hemolysis at 256 μg/ml values of the **Ru-3** was only 4%. Therefore, in terms of activity and toxicity, **Ru-3** exhibited the most promising antibacterial behavior among them. Herein, different auxiliary ligands affect the physicochemical properties of the complexes, especially hydrophilicity and lipophilicity, which were considered to be closely related to the antibacterial ability (Chopra et al., 2015). The distribution coefficient data show that **Ru-3** (logD o/w of ca. 1.7885) displayed more apparent lipophilicity than **Ru-1** (logD o/w of ca. 1.1445) and **Ru-2** (logD o/w of ca. 0.9681). Meanwhile, complexes with two positive charges are favorable for the interaction with negatively charged substances on the bacterial cell membrane, and further lead to destroy the membrane and cause bacterial death. Therefore, herein the polarity of **Ru-3** containing 4,4 ′- dimethyl-2,2′- bipyridine ligands probably contribute to the antibacterial activity against *Staphylococcus aureus*.

**FIGURE 3:**
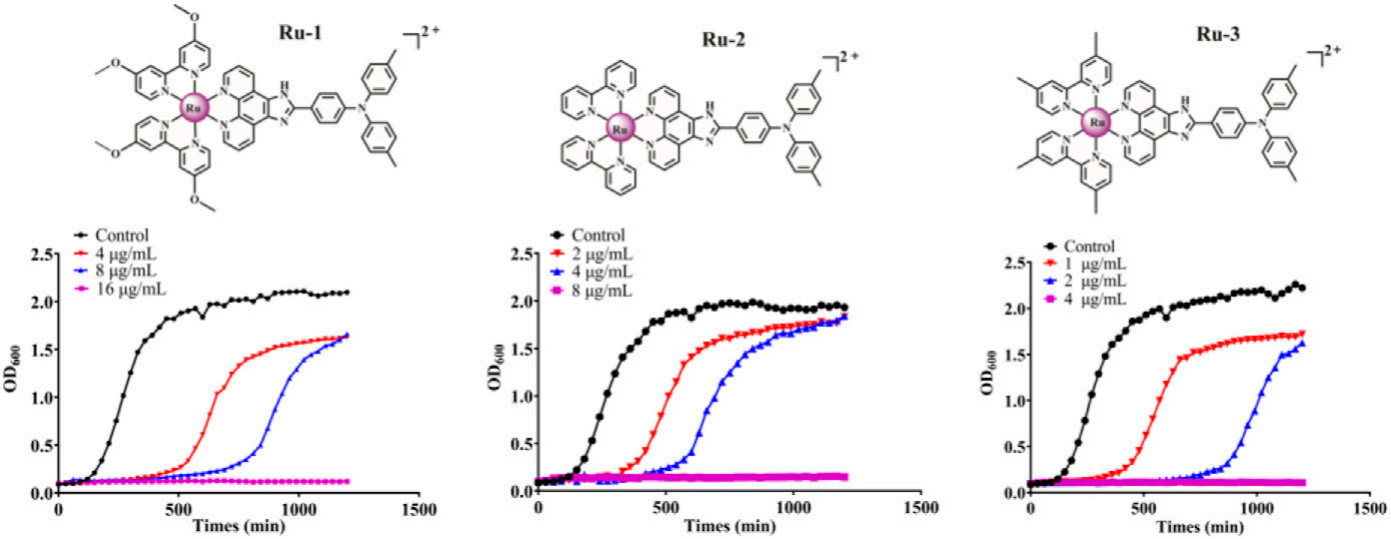
The inhibitory effect of Ru-1, Ru-2 and Ru-3 on *Staphylococcus aureus*. The bacterial culture was cultured in a plate reader at 37°C with orbit shaking at 180 rpm. The OD_600_ was recorded at 30 min intervals.

**FIGURE 4:**
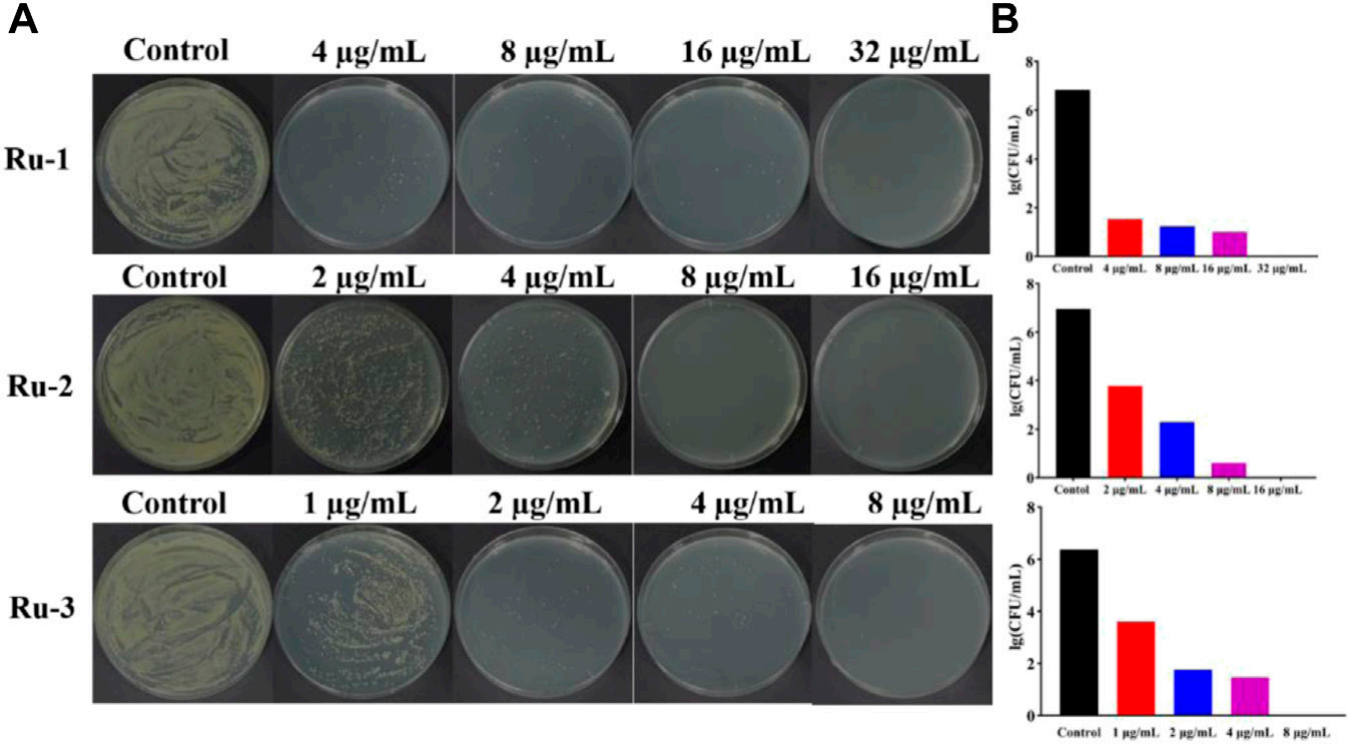
Ruthenium complex inhibited the growth of wild-type *Staphylococcus aureus* for 2 h **(A)** the plate experiment diagram. **(B)** the plate counting diagram.

Then, the effects of ruthenium complexes on the growth of *Staphylococcus aureus* was explored by measuring the growth curve of *Staphylococcus aureus*. As shown in Figure 3, the growth curve of three Ru complexes showed a dose-dependent inhibitory effect on *Staphylococcus aureus*. Further quantitative analysis was carried out by colony forming units (CFU) on agar plate to evaluate the activity of the complexes against *Staphylococcus aureus*. As shown in Figure 4, the antibacterial behaviors of **Ru-1**—**Ru-3** were obviously dose-dependent, and **Ru-3** still showed the best antibacterial effect among the three complexes, which was consistent with the experimental results of MIC value determination above.

### Inhibit biofilm formation and bacterial biofilm disruption

**FIGURE 5:**
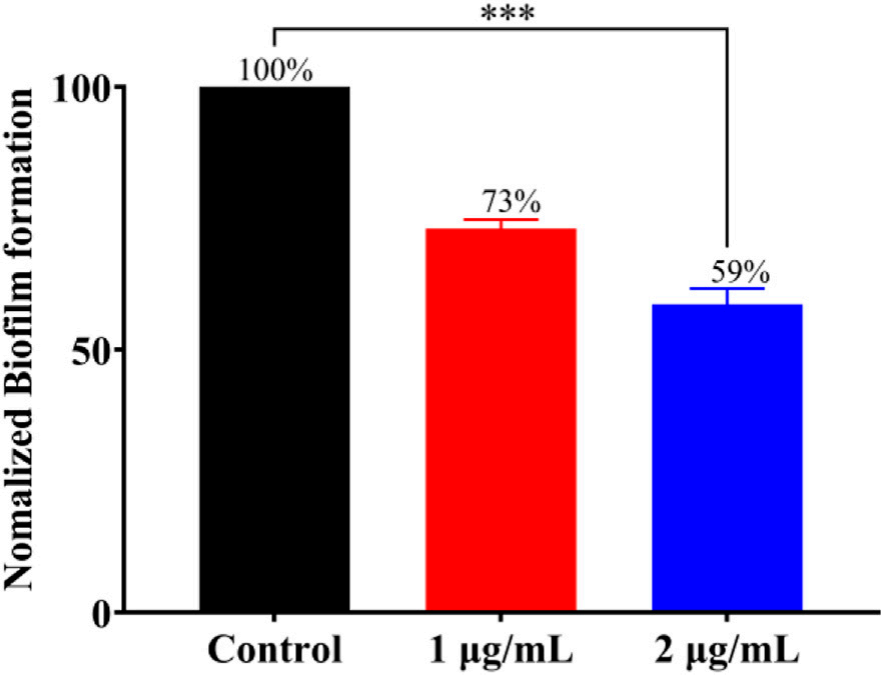
The effect of Ru-3 (1 μg ml^−1^ and 2 μg ml^−1^) on the biofilm formation of *Staphylococcus aureus*.

Bacterial biofilm is a viscous structure formed by bacterial aggregation, which can protect bacterial cells from external influence and effectively resist the action of antibiotics (Chopra et al., 2015), and more than 80% of clinically malignant infections are associated with bacterial biofilm resistance (Conti et al., 2021). According to the above experiments, **Ru-3** was proved as the most effective complex. To further explore whether this complex can inhibit the formation of *S. aureus* biofilm, crystal violet staining method was carried out. **Ru-3** of sub inhibitory concentration was performed to ensure that it affected the formation of biofilm rather than kill bacteria. As shown in Figure 5, biofilm formation in the presence of **Ru-3** was significantly reduced by 27% and 41% at the concentration of 1 μg ml^−1^ and 2 μg ml^−1^, respectively. These results indicated that **Ru-3** can obviously inhibit the formation of biofilm at sub inhibitory concentratidon.

**FIGURE 6:**
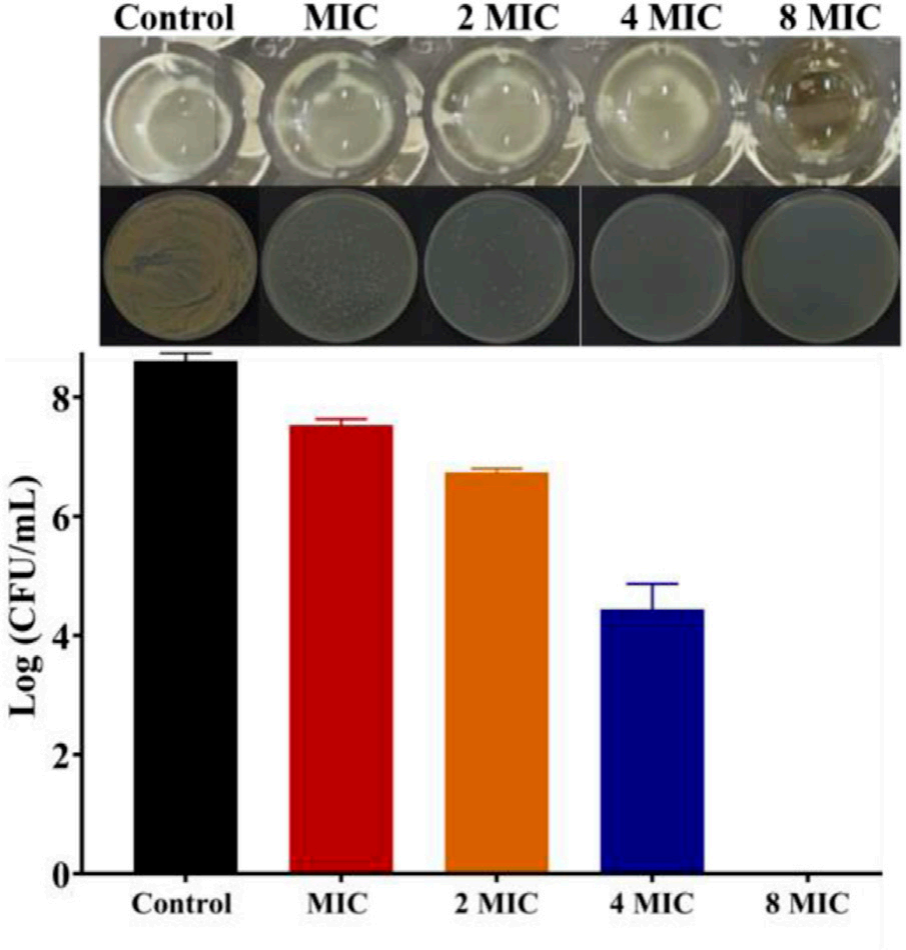
The results of killing the *Staphylococcus aureus* in the biofilm and count results of killing *Staphylococcus aureus* in biofilm.

Then, bacterial biofilm destruction assay was performed to further explore whether **Ru-3** can destroy biofilm, and the results were shown in Figure 6. It was clear that **Ru-3** displayed a significant effect on killing *Staphylococcus aureus* in biofilm, and with the increase of **Ru-3** concentration, the number of living bacteria in biofilm decreased dramatically. The number of the survival *Staphylococcus aureus* in the biofilm decreased from the initial 8.62 to 0 log10 colony-forming units per milliliter (CFU/ml) as the concentration of **Ru-3** was 32 μg ml^−1^, which was exactly same as its MBC value. These data indicated that **Ru-3** can inhibit not only the formation of biofilms, but also the already formed bacterial biofilms, suggesting that the antimicrobial behavior of **Ru-3** probably not be prone to drug resistance.

### Resistance study

**FIGURE 7:**
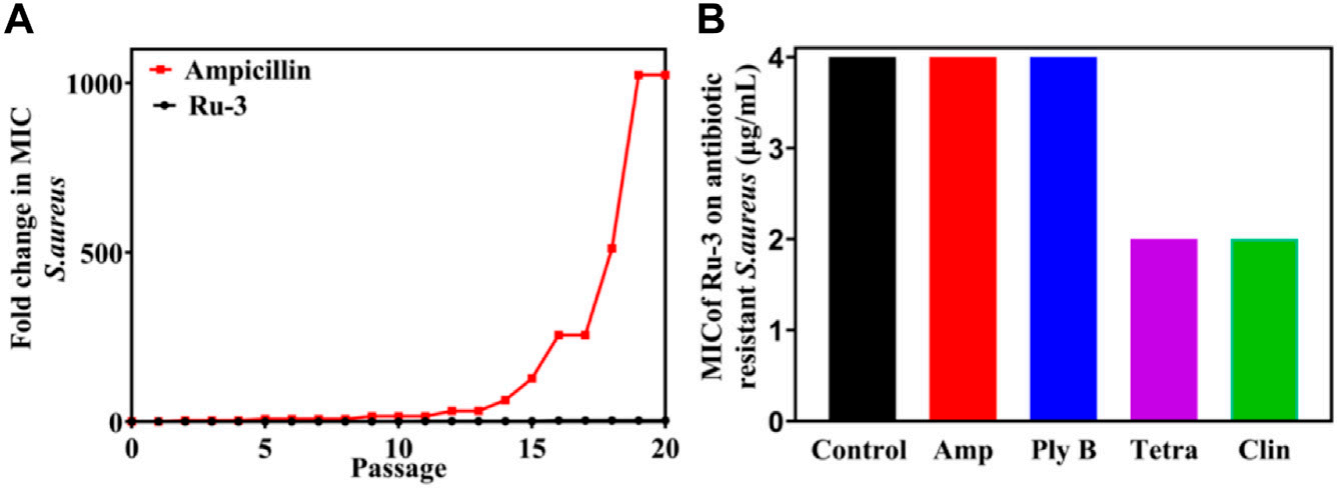
Bacterial resistance study of compound Ru-3 against *S. aureus* (Ampicillin was selected as a reference drug). **(A)** Fold changes in MIC values of compound Ru-3 and ampicillin against *Staphylococcus aureus*. **(B)** MIC of Ru-3 on different antibiotic resistant *Staphylococcus aureus*. Control: Wild *Staphylococcus aureus*. Amp: Ampicillin resistant *Staphylococcus aureus*. Ply **(B)** Polymyxin B resistant *Staphylococcus aureus*. Tetra: tetracycline resistant *Staphylococcus aureus*. Clin: Clindamycin resistant *Staphylococcus aureus*.

Antibiotic resistance has become an increasingly serious problem (Laxminarayan et al., 2020). To study the possibility of drug resistance induced by **Ru-3**, the drug resistance of *Staphylococcus aureus* for 20 generations was carried out at sublethal concentration. After 20 generations of *Staphylococcus aureus* culture, the MIC value of **Ru-3** increased only four times, indicating that **Ru-3** was not easy to be resistant to *Staphylococcus aureus*. In contrast, the lactam antibiotic ampicillin sodium rapidly induced bacterial drug resistance, and the MIC value increased more than 1024 times after 20 passages (Figure 7A) under the same experimental conditions. This result probably related to the rapid bactericidal effect of **Ru-3** and the destruction of bacterial cell membrane. More importantly, **Ru-3** unexpectedly exhibited apparent antibacterial activity against antibiotics resistant *Staphylococcus aureus*, which were obtained by treating with a variety of antibiotics for 20 generations (Figure 7B). The results showed that the MIC values of **Ru-3** against antibiotic resistant *S. aureus* were almost the same as the wild type *Staphylococcus aureus*. All the results showed that **Ru-3** had strong antibacterial activity against antibiotic resistant bacteria and had no obvious drug resistance.

### Hemolysis test

**FIGURE 8:**
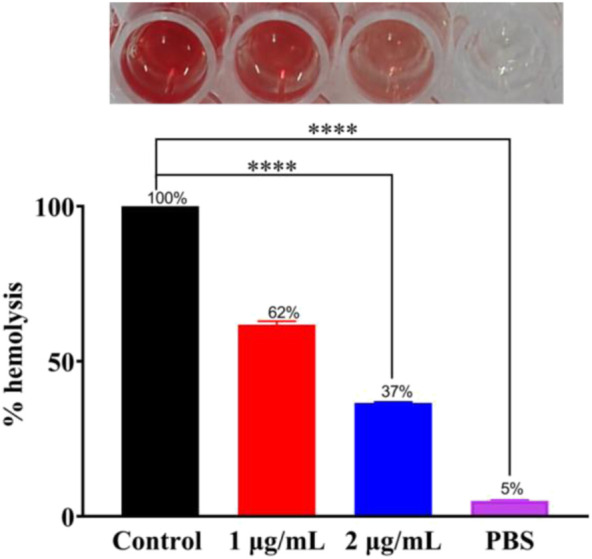
Experimental results of Ru-3 inhibitory toxin. Effect of 1 μg ml^−1^ or 2 μg ml^−1^ Ru-3 on the secretion of *Staphylococcus aureus* hemolysin. All experiments had three biological replicates.

The toxin produced by bacteria is also one of the primary causes of disease. To find whether **Ru-3** can inhibit the produced toxin by *Staphylococcus aureus*, hemolysis test was carried out. As shown in Figure 8, after incubation for 24 h, there was barely different in OD_600_ values between 1 μg ml^−1^, 2 μg ml^−1^ and control group. Toxin was prominently reduced by 38% and 63% in the presence of **Ru-3** of 1 μg ml^−1^ and 2 μg ml^−1^ respectively. This showed that **Ru-3** could inhibit the toxin production of *Staphylococcus aureus* at sub inhibitory concentration. To verify that the rupture was not caused by buffer (PBS), a sterile control was also performed. The results showed that the red blood cells remained intact, indicating that the rupture of red blood cells was caused by the secretion of hemolysin by bacteria, and **Ru-3** effectively inhibited the secretion of hemolysin.

### Synergistic effects with antibiotics

**FIGURE 9:**
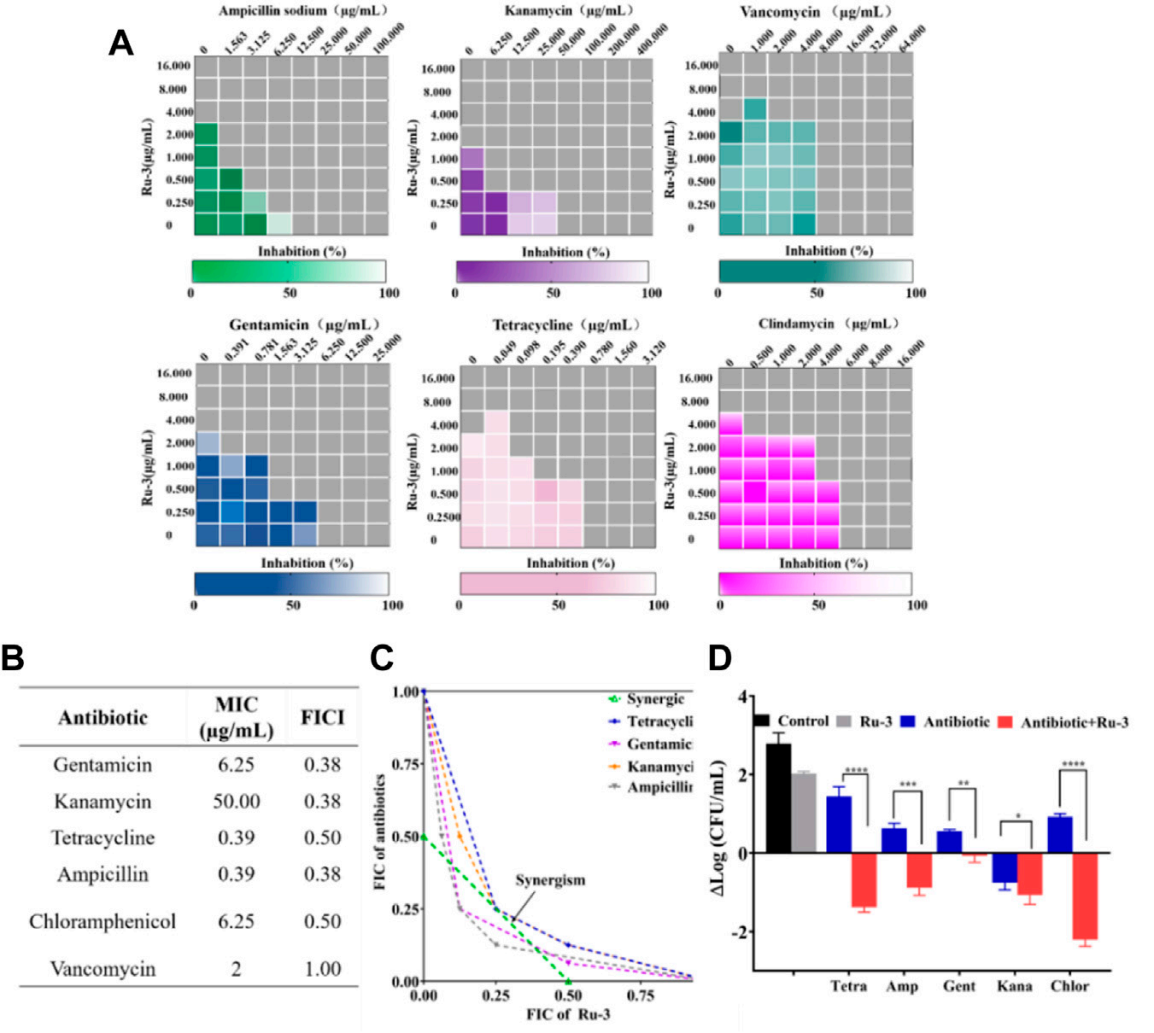
**(A)** Heat plots of checkerboard assays for Ru-3 in combination with different antibiotics against *Staphylococcus aureus*. **(B)** MIC of antibiotics and fractional inhibitory concentration indices (FIC) of the combination with Ru-3 against *Staphylococcus aureus* **(C)** An isobologram analysis of the synergistic effects of Ru-3 with Ampicillin, Chloramphenicol, Gentamicin, Kanamycin and Tetracyclines. **(D)** Logarithmic change of CFU mL^−1^ (from time zero) of *Staphylococcus aureus* after treatment with Ru-3 (2 μg ml^−1^) and antibiotics with combined effect (0.25 MIC) for 3 h.

Antimicrobial adjuvants are considered as magic weapons against drug resistant bacteria. To study whether **Ru-3** can also be used as antibacterial adjuvant, the interactions between **Ru-3** and common antibiotics were performed by checkerboard method (Zhang et al., 2022). Fractional inhibitory concentration index (FICI) is defined as the sum of the MIC of each drug when used in combination divided by the MIC of the drug when used alone (synergism (FICI ≤0.5), preparability (0.5 < FICI ≤1), no difference (1 < FICI ≤2), antagonistic (FICI >2)) (Chen et al., 2021). As shown in Figure 9A, **Ru-3** had synergistic effects with kanamycin, gentamicin, chloramphenicol, ampicillin sodium and tetracycline, which demonstrated that **Ru-3** possessed antibacterial synergistic effect on a variety of antibiotics. To further reveal that synergistic effect, the incubations of the above antibiotics (gentamicin, ampicillin sodium, kanamycin, chloramphenicol and tetracycline) and **Ru-3** of sublethal concentration (0.25 MIC) were used to treat *S. aureus*. (Figure 9D). As expected, the antibacterial activity was significantly enhanced, indicating that there were distinct synergistic effects between them. Therefore, **Ru-3** not only exhibited obvious direct antibacterial activity, but also was a potential antibacterial adjuvant, which can effectively increase antimicrobial activity of some existing antibiotics.

### Membrane damage of *Staphylococcus aureus*

The poor permeability of traditional antibiotics is one of the main reasons for the decrease of its therapeutic ability and the increase of multi drug resistant bacteria (Sun et al., 2021; Yan and Bassler, 2019; Gafur et al., 2020). Therefore, excellent membrane damage ability should be an important characteristic of newly developed antibacterial agents (Wang et al., 2019). To elucidate whether **Ru-3** can destroy the integrity of the bacterial membrane, the following experiments were carried out, including membrane depolarization studies, DAPI/PI staining and fluorescence microscope, ONPG experiment, leakage of nucleic acid and SEM.

**FIGURE 10:**
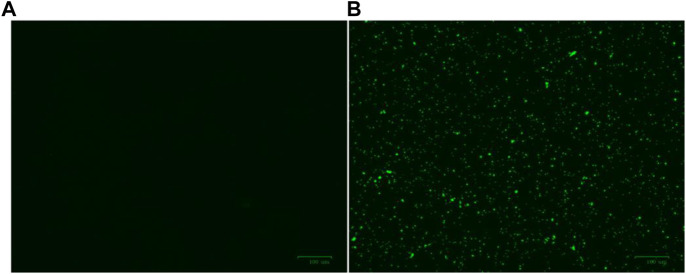
Effect of membrane depolarization **(A)** The blank control was bacteria without drug treatment. **(B)** *Staphylococcus aureus* treated with Ru-3 (4 μg ml^−1^) for 2 h.

Firstly, the membrane destruction ability of active molecule **Ru-3** was studied by fluorescence microscopy with 3, 3′-dipropylthiadicarbocyanine iodide [DiSC_3_(5)]. DiSC_3_ accumulates in cells on the polarized membrane, resulting in fluorescence self-quenching. However, when the integrity of the cell membrane is damaged by the change of membrane potential, DiSC_3_(5) will released from the cell membrane, resulting in a sharp increase in fluorescence intensity (Guo et al., 2021). As shown in Figure 10, the negative group showed no fluorescence in the stained group, while strong green fluorescence occurred in the **Ru-3** treated group. Indicating that compound **Ru-3** has effect on the bacterial cell membranes.

**FIGURE 11:**
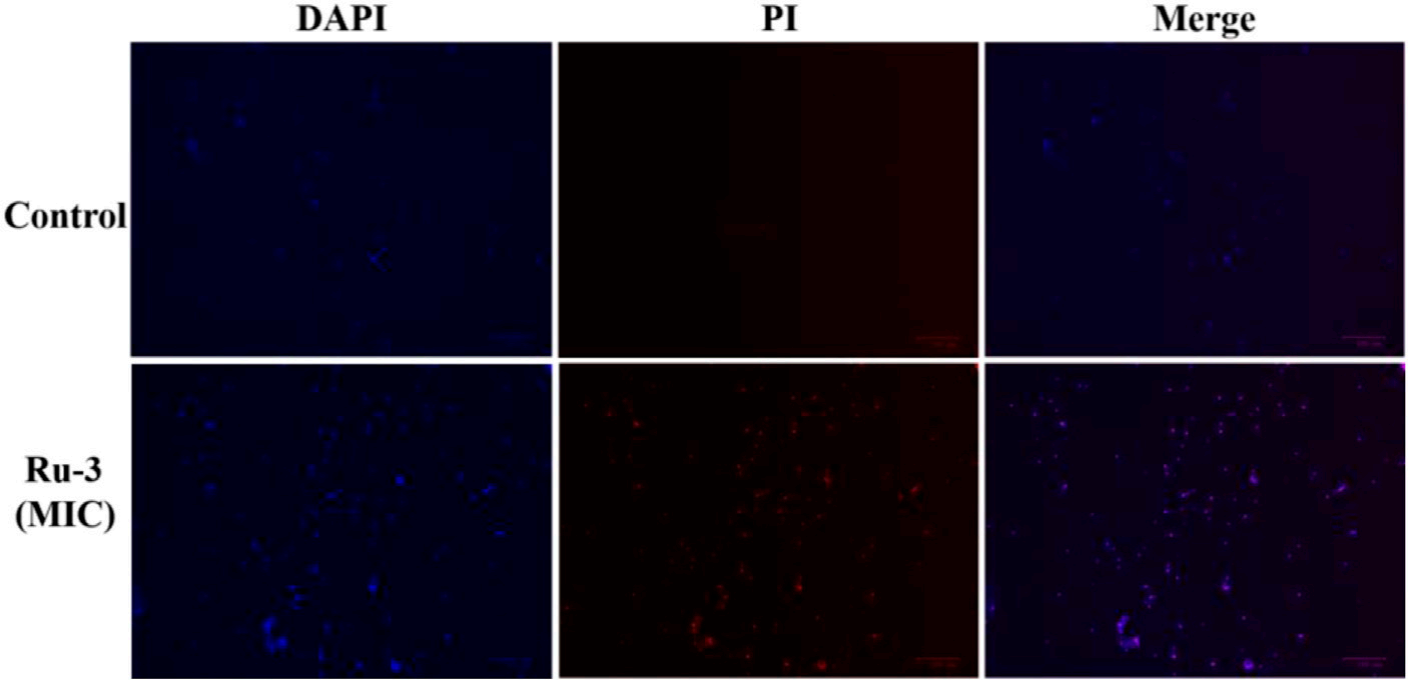
Fluorescence images of *Staphylococcus aureus* control or treated with Ru-3 (4 μg ml^−1^) for 2 h, which stained with DAPI, PI. Scale bar: 100 *μ*m.

Secondly, 4′, 6-diamino-2-phenylindole (DAPI) and propidium iodide (PI) as staining agents to further analyze the antibacterial mechanism of compound **Ru-3**. DAPI can enter both living and dead cells and produce blue fluorescence, while PI can only enter cells with damaged membrane and combine with nucleic acid to produce red fluorescence (Sun et al., 2021). As shown in Figure 11, only blue fluorescence was observed in the negative control group, suggesting intact cell membranes of *Staphylococcus aureus*. In contrast, blue and red fluorescence was observed for the **Ru-3** groups, indicating that **Ru-3** can effectively disintegrate *S. aureus* membrane.

**FIGURE 12:**
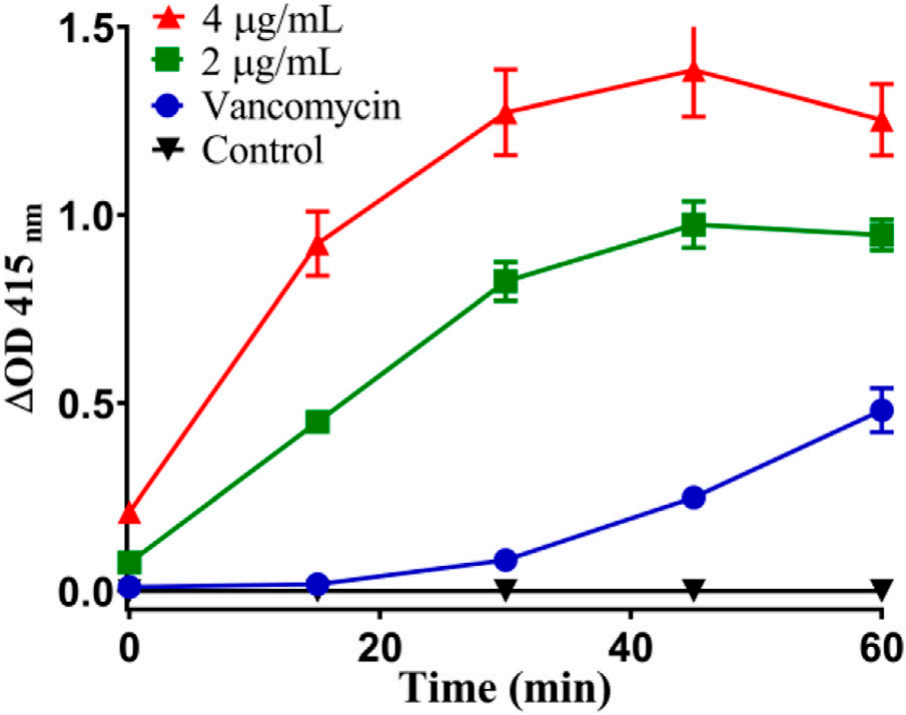
Plasma membrane permeability of *Staphylococcus aureus* cells treated with Ru-3 (2 μg/ml or 4 μg/ml) and vancomycin (2 μg/ml).

Thirdly, once the lipid bilayer of bacteria is physically destroyed, the cytoplasmic content will overflow (Rasamiravaka et al., 2015). When the cytoplasmic membrane is permeable, the non-permeable membrane chromogenic substrate o-nitrobenzene-β-Galactoside (ONPG) enters the cytoplasm and is destroyed by *β*-Galactosidase degraded to produce o-nitrophenol, showing special absorbance at 415 nm (Xuan et al., 2021). As shown in Figure 12, the plasma membrane permeability of *Staphylococcus aureus* induced by **Ru-3** increased with time and showed a concentration dependent trend. Compared with vancomycin, **Ru-3** exhibited better membrane permeability. The above results showed that the treatment of **Ru-3** caused damage to *Staphylococcus aureus* cells, which lead to the physical destruction of lipid bilayer and cell membrane, resulting in the serious leakage of cell contents.

**FIGURE 13:**
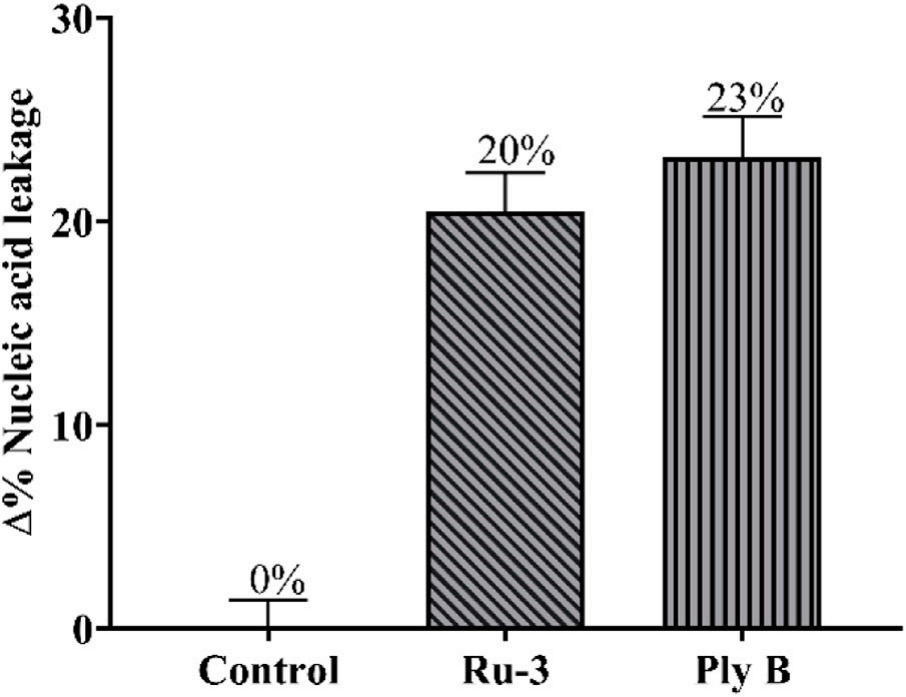
Percentage content of leaked nucleic acid from bacterial cells with the treatment of Ru-3 (4 μg/ml) or Ply B (Polymyxin B, 100 μg/ml).

Fourthly, the damage of **Ru-3** to the membrane was further verified by measuring the leakage of nucleic acid. Nucleic acids have a characteristic UV absorption at 260 nm, therefore, the degree of cell nucleic acid leakage can be evaluated by observing the change in absorbance at 260 nm of the bacterial solution (Cui et al., 2015). As shown in Figure 13, after treating *S. aureus* with ruthenium **Ru-3** or polymyxin B, nucleic acid leakage increased significantly from 0% to 20% and 23%, comparing with the blank. The above results demonstrated that the treatment of **Ru-3** caused damage to *Staphylococcus aureus* cell membrane, resulting in the leakage of intracellular proteins. Importantly, **Ru-3**’s ability to break through cell membranes was much better than polymyxin B, which was a typical antibiotic that disrupted bacterial membrane.

**FIGURE 14:**
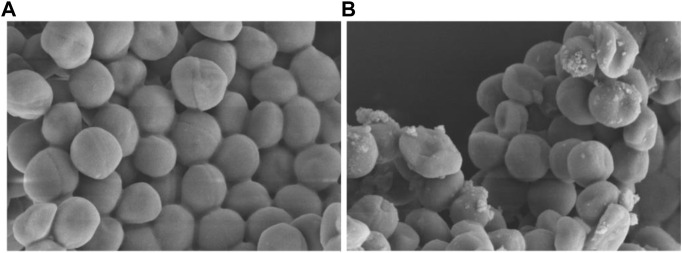
Scanning electron microscope (SEM) images of the cell membrane of *Staphylococcus aureus* cells treated with PBS or Ru-3 **(A)** PBS. **(B)** Ru-3 (4 μg/ml).

Finally, the morphological observation of bacterial samples provides distinct evidence for the destruction and damage of bacterial cell membrane. As shown in Figures 14A,B, it was obvious that the bacteria in the control group showed a complete and smooth cell surface without rupture. After the treatment with **Ru-3** of a concentration of 4 μg ml^−1^ for 2 h, most of the bacterial structures were deformed, collapsed and many ripples were observed, revealing that the treatment of **Ru-3** would lead to the physical destruction of cell membrane.

### Toxicity study

**FIGURE 15:**
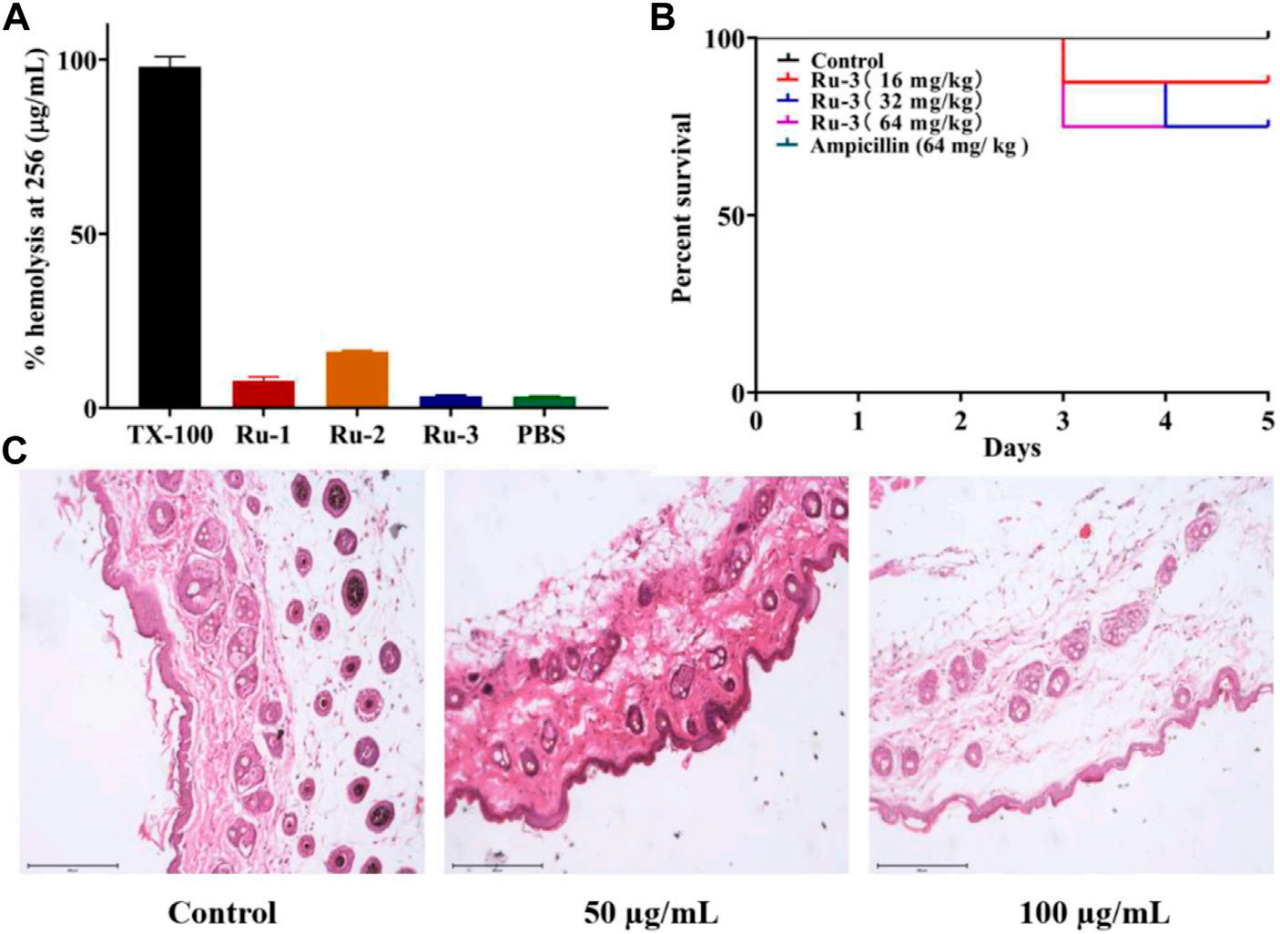
Test results of Ru compounds toxicity **(A)** % hemolysis at 256 μg ml^−1^ of Ru compounds. **(B)** Kaplan-Meier survival curves. Determination of Ru-3 toxicity in the insect model Galleria mellonella (Ampcillin’s curve coincides with control’s curve.) Larvae were injected with 5 µL of water (control), or Ru-3 (4–512 mg kg^−1^). The larvae were incubated at 37.5°C and live/dead scores were conducted at 120 h **(C)** H&E staining images of the infected tissues with different treatments.

Based on the excellent antibacterial effect of **Ru-3** *in vitro*, its biosafety was further evaluated. Firstly, the hemolytic activity of ruthenium complexes on rabbit red blood cells was measured to study their toxicity. As shown in Figure 15A, **Ru-3** displayed negligible hemolytic activity, even if the concentration was as high as 256 μg ml^−1^. In view of its good compatibility with mammalian red blood cells, the toxicity of **Ru-3** to eukaryotes was also studied. Herein, **Ru-3** was tested with Gallery melonella larvae. Because its physiology and immune system are extremely similar to mammals, this insect model is widely used as an *in vivo* model, especially in toxicity screening, which produces results comparable to more commonly used mammalian models (Roy et al., 2019). The results were shown in Figure 15B. When the concentration of **Ru-3** was 64 mg kg^−1^, the survival rate was still 75%. Therefore, it indicated that **Ru-3** has low toxicity and good biocompatibility.

Finally, the skin irritation of **Ru-3** on BALB/c mice was further studied. Hematoxylin eosin (H&E) staining was used to study the pathological changes of muscle tissue caused by **Ru-3** in Figure 15C (Weber et al., 2016). Comparing the images produced by the treated and untreated tissues, the tissue sections of the **Ru-3** treatment group were very similar to the normal mouse tissues, indicating that there were no obvious pathological abnormalities. Therefore, **Ru-3** can be considered as a non-irritating complex and has good antibacterial effect and biocompatibility.

### *In vivo* antibacterial assay

**FIGURE 16:**
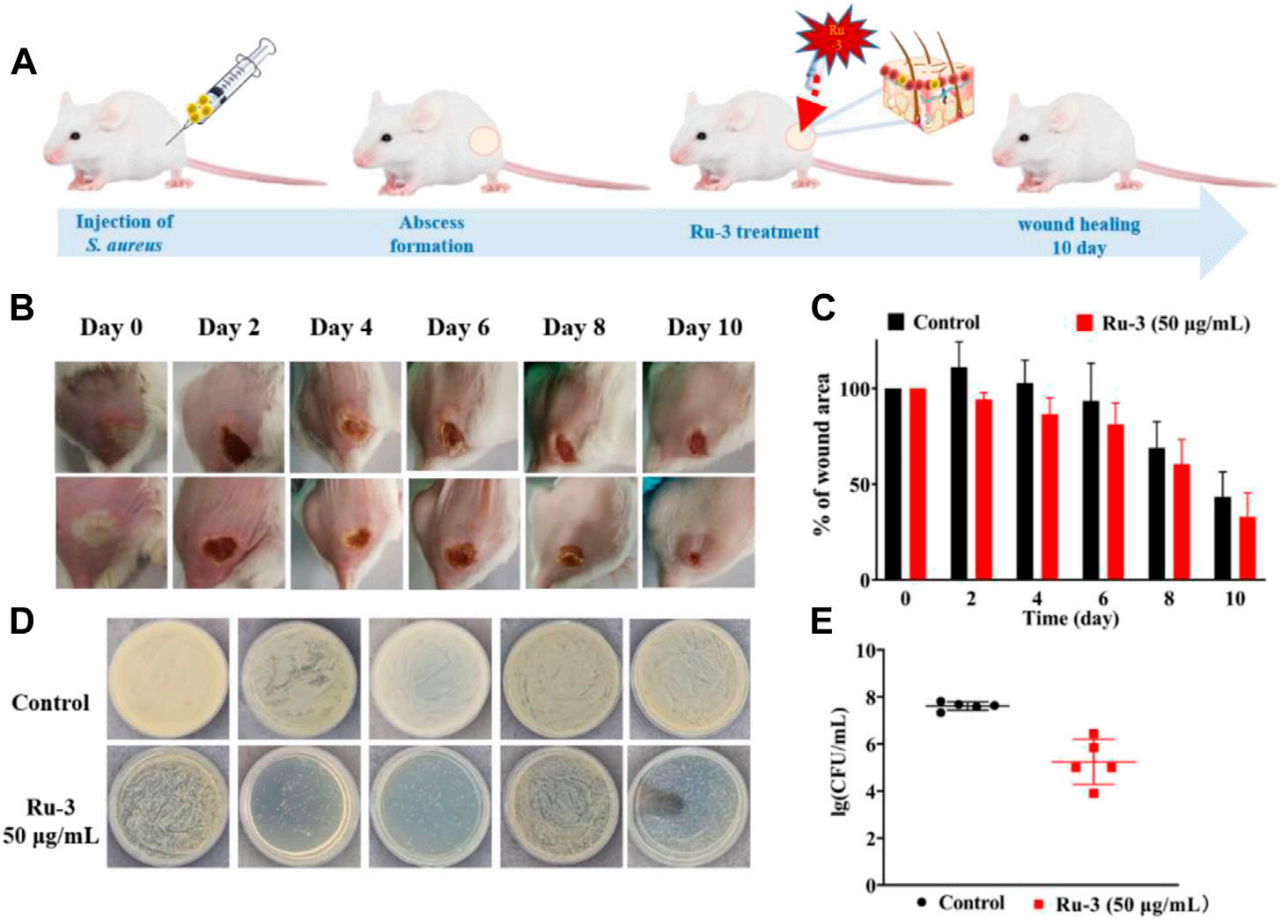
Treatment of *Staphylococcus aureus* skin infection *in vivo* **(A)** Scheme illustration of the procedures including the establishment of the *Staphylococcus aureus* infection mouse model and subsequent treatment regime. **(B)** Representative photos of skin abscesses with/without Ru-3 treatment **(C)** Change diagram of mouse wond. **(D)** Plate diagram of mouse wond colony calculation. **(E)** Colony count of wound in mice.

The above results have confirmed that **Ru-3** had good antibacterial activity against *Staphylococcus aureus*. To further explore whether **Ru-3** has significant antibacterial activity *in vivo*, a mouse skin infection model was established. The day before, the hair of the infected part of the mice was removed, then *Staphylococcus aureus* was inoculated to form an abscess on the skin. Subsequently, all mice were divided into two groups, and one group used cream with containing **Ru-3** (50 μg ml^−1^) and other group used only cream, applying 4 times a day to the abscess. As shown in Figure 16, photos of infected tissue were obtained after 10 days, and subsequent wound healing was used to determine its antibacterial activity. Figure 16A showed a schematic diagram of infection and treatment regimen, and Figure 16B shows changes in wound healing after **Ru-3** (50 μg ml^−1^, and untreated mice as control) treatment. Obviously, after 4 days of **Ru-3** treatment, the degree of wound healing increased significantly. Which indicates that **Ru-3** also has antibacterial activity *in vivo*.

## Conclusions

In conclusion, functionalized ruthenium complex with triphenylamine (TPA) had good antibacterial activity against *Staphylococcus aureus*. **Ru-3** inhibited the formation of biofilm at sublethal concentration and killed the bacteria in the formed biofilm at eight MIC. According to the results of fluorescence staining, ONPG, nucleic acid leakage and scanning electron microscope, it was found that the mechanism of **Ru-3** may be the destruction of bacterial cell membrane. It is exciting to find that **Ru-3** can effectively inhibit the secretion of hemolysin from *Staphylococcus aureus* and has a low rate of auto-hemolysis. More importantly, it has low toxicity and good biocompatibility to the great wax beetle whose physiology and immune system are surprisingly same to mammals. The joint sensitivity test shows that **Ru-3** has synergistic effect on a variety of commonly used antibiotics, and maintains the original MIC value for some antibiotic resistant bacteria, which is helpful to formulate clinical medication plan. Finally, the *in vivo* infection study on mice showed that **Ru-3** significantly improved the wound healing process after skin infection with bacteria, and had no irritating effect on the skin. Obviously, multifunctional Ru complexes modified with TPA have great potential for the development of anti *Staphylococcus aureus* agents.

## Data Availability

The datasets presented in this study can be found in online repositories. The names of the repository/repositories and accession number(s) can be found below: https://www.ccdc.cam.ac.uk/solutions/csd-system/components/csd/.

## References

[B1] Abouelhassan Y. Garrison A. T. Yang H. Chávez-Riveros A. Burch G. M. Huigens R. W. (2019). Recent progress in natural-product-inspired programs aimed to address antibiotic resistance and tolerance. J. Med. Chem. 62, 7618–7642. 10.1021/acs.jmedchem.9b00370 30951303 PMC6742553

[B2] Aslam B. Wang W. Arshad M. I. Khurshid M. Muzammil S. Rasool M. H. (2018). Antibiotic resistance: a rundown of a global crisis. Infect. Drug Resist. 11, 1645–1658. 10.2147/IDR.S173867 30349322 PMC6188119

[B3] Carlsen P. H. J. Katsuki T. Martin V. S. Sharpless K. B. (1981). A greatly improved procedure for ruthenium tetroxide catalyzed oxidations of organic compounds. J. Org. Chem. 46, 3936–3938. 10.1021/jo00332a045

[B4] Castellano F. N. Dattelbaum J. D. Lakowicz J. R. (1998). Long-lifetime Ru(II) complexes as labeling reagents for sulfhydryl groups. Anal. Biochem. 255, 165–170. 10.1006/abio.1997.2468 9451499

[B5] Chen J. P. Battini N. Ansari M. F. Zhou C. H. (2021). Membrane active 7-thiazoxime quinolones as novel DNA binding agents to decrease the genes expression and exert potent anti-methicillin-resistant *Staphylococcus aureus* activity. Eur. J. Med. Chem. 217, 113340. 10.1016/j.ejmech.2021.113340 33725630

[B6] Chopra L. Singh G. Jena K. K. Sahoo D. K. (2015). Sonorensin: A new bacteriocin with potential of an anti-biofilm agent and a food biopreservative. Sci. Rep. 5, 13412. 10.1038/srep13412 26292786 PMC4544038

[B7] Collin J. P. Sauvage J. P. (1986). Synthesis and study of mononuclear ruthenium(II) complexes of sterically hindering diimine chelates. Implications for the catalytic oxidation of water to molecular oxygen. Inorg. Chem. 25, 135–141. 10.1021/ic00222a008

[B8] Conti L. Mengoni A. Giacomazzo G. E. Mari L. Perfetti M. Fagorzi C. (2021). Exploring the potential of highly charged Ru(II)- and heteronuclear Ru(II)/Cu(II)-polypyridyl complexes as antimicrobial agents. J. Inorg. Biochem. 220, 111467. 10.1016/j.jinorgbio.2021.111467 33932708

[B9] Cui H. Y. Zhao C. T. Lin L. (2015). Antibacterial activity of Helichrysum italicum oil on VegeTables and its mechanism of action. J. Food Process. Preserv. 39, 2663–2672. 10.1111/jfpp.12516

[B10] De Oliveira D. M. P. Kidd B. M. Harris P. N. A. Beatson M. A. Beatson S. A. Paterson D. L. (2020). Antimicrobial resistance in ESKAPE pathogens. Clin. Microbiol. Rev. 33, 001811–e219. 10.1128/cmr.00181-19 PMC7227449 32404435

[B11] Gafur A. Sukamdani G. Y. Kristi N. Maruf A. Xu J. Chen X. (2020). From bulk to nano-delivery of essential phytochemicals: recent progress and strategies for antibacterial resistance. J. Mater. Chem. B 8, 9825–9835. 10.1039/d0tb01671c 33000844

[B12] Gitlin J. D. Lill R. (2012). Special issue: Cell biology of metals. Biochim. Biophys. Acta (BBA) - Mol. Cell Res. 9, 1405–1642. 10.1016/j.bbamcr.2012.07.008

[B13] Gorle A. K. Feterl M. Warner J. M. Wallace L. Keene F. R. Collins J. G. (2014). Tri- and tetra-nuclear polypyridyl ruthenium(II) complexes as antimicrobial agents. Dalton Trans. 43, 16713–16725. 10.1039/c4dt02139h 25271478

[B14] Guo Y. Hou E. H. Wen T. Y. Yan X. T. Han M. Y. Bai L. P. (2021). Development of membrane-active honokiol/magnolol amphiphiles as potent antibacterial agents against methicillin-resistant *Staphylococcus aureus* (MRSA). J. Med. Chem. 64, 12903–12916. 10.1021/acs.jmedchem.1c01073 34432450

[B15] Howerton B. S. Heidary D. K. Glazer E. C. (2012). Strained ruthenium complexes are potent light-activated anticancer agents. J. Am. Chem. Soc. 134, 8324–8327. 10.1021/ja3009677 22553960

[B16] Hussain S. Joo J. Kang J. Kim B. Braun G. B. She Z. G. (2018). Antibiotic-loaded nanoparticles targeted to the site of infection enhance antibacterial efficacy. Nat. Biomed. Eng. 2, 95–103. 10.1038/s41551-017-0187-5 29955439 PMC6015743

[B17] Kang M. M. Zhou C. C. Wu S. M. Yu B. G. Zhang Z. J. Song N. (2019). Evaluation of structure-function relationships of aggregation-induced emission luminogens for simultaneous dual applications of specific discrimination and efficient photodynamic killing of gram-positive bacteria. J. Am. Chem. Soc. 141, 16781–16789. 10.1021/jacs.9b07162 31553608

[B18] Knoll J. D. Turro C. (2015). Control and utilization of ruthenium and rhodium metal complex excited states for photoactivated cancer therapy. Coord. Chem. Rev. 282, 110–126. 10.1016/j.ccr.2014.05.018 25729089 PMC4343038

[B19] Laxminarayan R. Boeckel T. V. Frost I. Kariuki S. Khan E. A. Limmathurotsakul D. (2020). The lancet infectious diseases commission on antimicrobial resistance: 6 years later. Lancet Infect. Dis. 20, e51–e60. 10.1016/s1473-3099(20)30003-7 32059790

[B20] Lei X. L. Qiu L. Lan M. Du X. C. Zhou S. W. Cui P. F. (2020). Antibacterial photodynamic peptides for staphylococcal skin infection. Biomater. Sci. 8, 6695–6702. 10.1039/d0bm01467b 33108416

[B21] Li F. F. Collins J. G. Keene F. R. (2015). Ruthenium complexes as antimicrobial agents. Chem. Soc. Rev. 44, 2529–2542. 10.1039/c4cs00343h 25724019

[B22] Li J. Q. Meng Z. J. Zhuang Z. Y. Wang B. N. Dai J. Feng G. X. (2022). Effective therapy of drug-resistant bacterial infection by killing planktonic bacteria and destructing biofilms with cationic photosensitizer based on phosphindole oxide. Small 18, 2200743. 10.1002/smll.202200743 35347841

[B23] Liao X. W. Jiang G. J. Wang J. T. Duan X. M. Liao Z. Y. Lin X. L. (2020). Two ruthenium polypyridyl complexes functionalized with thiophen: Synthesis and antibacterial activity against *Staphylococcus aureus* . New J. Chem. 44, 17215–17221. 10.1039/d0nj02944k

[B24] Liu J. G. Zhang Q. L. Shi X. F. Ji L. N. (2001). Interaction of [Ru(dmp)2(dppz)]^2+^ and [Ru(dmb)_2_(dppz)]^2+^ with DNA: Effects of the ancillary ligands on the DNA-binding behaviors. Inorg. Chem. 40, 5045–5050. 10.1021/ic001124f 11531457

[B25] Liu S. S. Wang B. N. Yu Y. W. Liu Y. B. Zhuang Z. Y. Zhao Z. J. (2022). Cationization-enhanced type I and type II ROS generation for photodynamic treatment of drug-resistant bacteria. ACS Nano 16, 9130–9141. 10.1021/acsnano.2c01206 35584060

[B26] Mesquita M, Q. Dias C. J. Neves M. P. M. S. Almeida A. Faustino M. F. (2018). Revisiting current photoactive materials for antimicrobial photodynamic therapy. Molecules 23, 2424. 10.3390/molecules23102424 PMC6222430 30248888

[B27] Moumita M. j. Sourav A. Indira B. Arnab G. Arindam M. (2021). Effect of an imidazole-containing schiff base of an aromatic sulfonamide on the cytotoxic efficacy of N, N-coordinated half- sandwich ruthenium(II) p-cymene complexes. Inorg. Chem. 60, 4744–4754. 10.1021/acs.inorgchem.0c03706 33760599

[B28] Nyawade E. A. Friedrich H. B. Omondi B. Mpungose P. (2015). Synthesis and characterization of new (η5-Cyclopentadienyl) dicarbonyl ruthenium(II) amine complexes: Their application as homogeneous catalysts in styrene oxidation. Organometallics 34, 4922–4931. 10.1021/acs.organomet.5b00564

[B29] Patra M. Gasser G. Metzler-Nolte N. (2012). Small organometallic compounds as antibacterial agents. Dalton Trans. 41, 6350–6358. 10.1039/c2dt12460b 22411216

[B30] Peng Y. B. Tao C. Tan C. P. Zhao P. (2021). Mitochondrial targeted rhodium(III) complexes: Synthesis, characterized and antitumor mechanism investigation. J. Inorg. Biochem. 218, 111400. 10.1016/j.jinorgbio.2021.111400 33684684

[B31] Piddock L. J. V. (2016). Reflecting on the final report of the O’neill review on antimicrobial resistance. Lancet Infect. Dis. 16, 767–768. 10.1016/s1473-3099(16)30127-x 27208976

[B32] Rasamiravaka T. Labtani Q. Duez P. Jaziri M. E. (2015). the formation of biofilms by *Pseudomonas aeruginosa*: a review of the natural and synthetic compounds interfering with control mechanisms. Biomed. Res. Int. 2015, 1–17. 10.1155/2015/759348 PMC4383298 25866808

[B33] Richter M. F. Hergenrother P. J. (2019). The challenge of converting gram-positive-only compounds into broad-spectrum antibiotics. Ann. N. Y. Acad. Sci. 1435, 18–38. 10.1111/nyas.13598 29446459 PMC6093809

[B34] Roy S. Mondal A. Yadav V. Sarkar A. Banerjee R. Sanpui P. (2019). Mechanistic insight into the antibacterial activity of chitosan exfoliated MoS_2_ nanosheets: Membrane damage, metabolic inactivation, and oxidative stress. ACS Appl. Bio Mater. 2, 2738–2755. 10.1021/acsabm.9b00124 35030809

[B35] Sheldrick G. M. (2015). Crystal structure refinement with SHELXL. Acta Crystallogr. C Struct. Chem. C71, 3–8. 10.1107/s2053229614024218 PMC4294323 25567568

[B36] Singh A. Barman P. (2021). Recent advances in schiff base ruthenium metal complexes: Synthesis and applications. Top. Curr. Chem. (Cham). 379, 29–100. 10.1007/s41061-021-00342-w 34109453

[B37] Singha K. Laha P. Chandra F. Dehury N. Koner A. L. Patra S. (2017). Long-lived polypyridyl based mononuclear ruthenium complexes: Synthesis, structure, and azo dye decomposition. Inorg. Chem. 56, 6489–6498. 10.1021/acs.inorgchem.7b00536 28509536

[B38] Song M. Liu Y. Huang X. Ding S. Wang Y. Shen J. (2020). A broad-spectrum antibiotic adjuvant reverses multidrug-resistant Gram-negative pathogens. Nat. Microbiol. 5, 1040–1050. 10.1038/s41564-020-0723-z 32424338

[B39] Srivastava P. Shukla M. Kaul G. Chopra S. Patra A. K. (2019). Rationally designed curcumin based ruthenium(II) antimicrobials effective against drug-resistant *Staphylococcus aureus* . Dalton Trans. 48, 11822–11828. 10.1039/c9dt01650c 31215556

[B40] Sullivan B. P. Salmon D. J. J. Meyer T. (1978). Mixed phosphine 2, 2'-bipyridine complexes of ruthenium. Inorg. Chem. 17, 3334–3341. 10.1021/ic50190a006

[B41] Sun H. Huang S. Y. Jeyakkumar P. Cai G. X. Fang B. Zhou C. H. (2021). Natural berberine-derived azolyl ethanols as new structural antibacterial agents against drug-resistant *Escherichia coli* . J. Med. Chem. 65, 436–459. 10.1021/acs.jmedchem.1c01592 34964345

[B42] Sun W. Z. Jian Y. Zhou M. X. Yao Y. S. Tian N. Li C. (2021). Selective and efficient photoinactivation of intracellular *Staphylococcus aureus* and MRSA with little accumulation of drug resistance: Application of a Ru(II) complex with photolabile ligands. J. Med. Chem. 64, 7359–7370. 10.1021/acs.jmedchem.0c02257 34032114

[B43] Tacconelli E. Carrara E. Savoldi A. Harbarth S. Mendelson M. Monnet D. L. (2018). Discovery, research, and development of new antibiotics: The WHO priority list of antibiotic-resistant bacteria and tuberculosis. Lancet Infect. Dis. 18, 318–327. 10.1016/s1473-3099(17)30753-3 29276051

[B44] Tran N. H. Nguyen D. Dwaraknath S. Mahadevan S. Chavez G. Chavez A. (2013). An efficient light-driven P450 BM3 biocatalyst. J. Am. Chem. Soc. 39, 14484–14487. 10.1021/ja409337v PMC3938315 24040992

[B45] Varney A. M. Smitten K. L. Thomas J. A. McLean S. (2021). Transcriptomic analysis of the activity and mechanism of action of a ruthenium(II)-Based antimicrobial that induces minimal evolution of pathogen resistance. ACS Pharmacol. Transl. Sci. 4, 168–178. 10.1021/acsptsci.0c00159 33615170 PMC7887750

[B46] Wang L. L. Battini N. Bheemanaboina R. R. Y. Ansari M. F. Chen J. P. Xie Y. P. (2019). A new exploration towards aminothiazolquinolone oximes as potentially multi-targeting antibacterial agents: Design, synthesis and evaluation acting on microbes, DNA, HSA and topoisomerase IV. Eur. J. Med. Chem. 179, 166–181. 10.1016/j.ejmech.2019.06.046 31254919

[B47] Weber D. K. Sani M. A. Downton M. T. Keene F. R. Collins J. G. (2016). Membrane insertion of a dinuclear polypyridyl ruthenium(II) complex revealed by solid-state NMR and molecular dynamics simulation-implications for selective antibacterial activity. J. Am. Chem. Soc. 138, 15267–15277. 10.1021/jacs.6b09996 27786471

[B48] Xuan T. F. Wang Z. Q. Liu J. Yu H. T. Lin Q. W. Chen W. M. (2021). Design and synthesis of novel c-di-GMP G-quadruplex inducers as bacterial biofilm inhibitors. J. Med. Chem. 64, 11074–11089. 10.1021/acs.jmedchem.1c00465 34323486

[B49] Yan J. Bassler B. L. (2019). Surviving as a community: antibiotic tolerance and persistence in bacterial biofilms. Cell Host Microbe 26, 15–21. 10.1016/j.chom.2019.06.002 31295420 PMC6629468

[B50] Yang H. F. Kundra S. Chojnacki M. Liu K. Fuse M. A. Abouelhassan Y. (2021). A modular synthetic route involving N-Aryl-2-nitrosoaniline intermediates leads to a new series of 3-substituted halogenated phenazine antibacterial agents. J. Med. Chem. 64, 7275–7295. 10.1021/acs.jmedchem.1c00168 33881312 PMC8192493

[B51] Yu J. H. Xu X. F. Hou W. Meng Y. Huang M. Y. Lin J. (2021). Synthetic cajaninstilbene acid derivatives eradicate methicillin-resistant *Staphylococcus aureus* persisters and biofilms. Eur. J. Med. Chem. 224, 113691. 10.1016/j.ejmech.2021.113691 34274830

[B52] Zhang H. G. Tao X. T. Chen K. S. Yuan C. X. Yan S. N. Jiang M. H. (2011). Off–on–off luminescent switching of a dye containing imidazo [4, 5-f] [1, 10]phenanthroline. Chin. Chem. Lett. 22, 647–650. 10.1016/j.cclet.2010.12.005

[B53] Zhang Q. Xiong Y. S. Cheng J. X. Tan Y. H. Liao X. W. Wang J. T. (2022). Synthesis and biological evaluation of ruthenium polypyridine complexes with 18β-glycyrrhetinic acid as antibacterial agents against *Staphylococcus aureus* . Dalton Trans. 51, 1099–1111. 10.1039/D1DT02692E 34935812

