# Supplementary material for: Ruthenium polypyridine complexes with triphenylamine groups as antibacterial agents against Staphylococcus aureus with membrane-disruptive mechanism
Source: Front Chem. 2022 Oct 10;10:1035741. doi: 10.3389/fchem.2022.1035741 (PMC9589286; doi:10.3389/fchem.2022.1035741)
Supplement: Supplementary file 1 [file DataSheet1.docx]

Supplementary Material

## List of contents

The synthetic route of ligand and ruthenium complexes...........................S1

three ruthenium complexes’ UV-Vis spectral data in 1-octanol………….S2

Crystal structure diagram...........................................................................S3

Killing of *S. aureus* strain by ruthenium complex………………………..S4

^1^H NMR, ^13^C NMR and HRMS for the complexes (**Ru-1**–**Ru-3**)………...S5-S15

HRMS spectrum of complexes……………………………………………S16

Stability of three compounds………………………………………………S17

Crystallographic data of **Ru-3** …...………………………………………Table S1

Selected bond lengths [Å] **Ru-3** and Selected angles (°) for **Ru-3**…………TableS2





**FIGURE. S1.** Synthetic route.





**FIGURE. S2.** Three ruthenium complexes’ UV-Vis spectral data in 1-octanol.


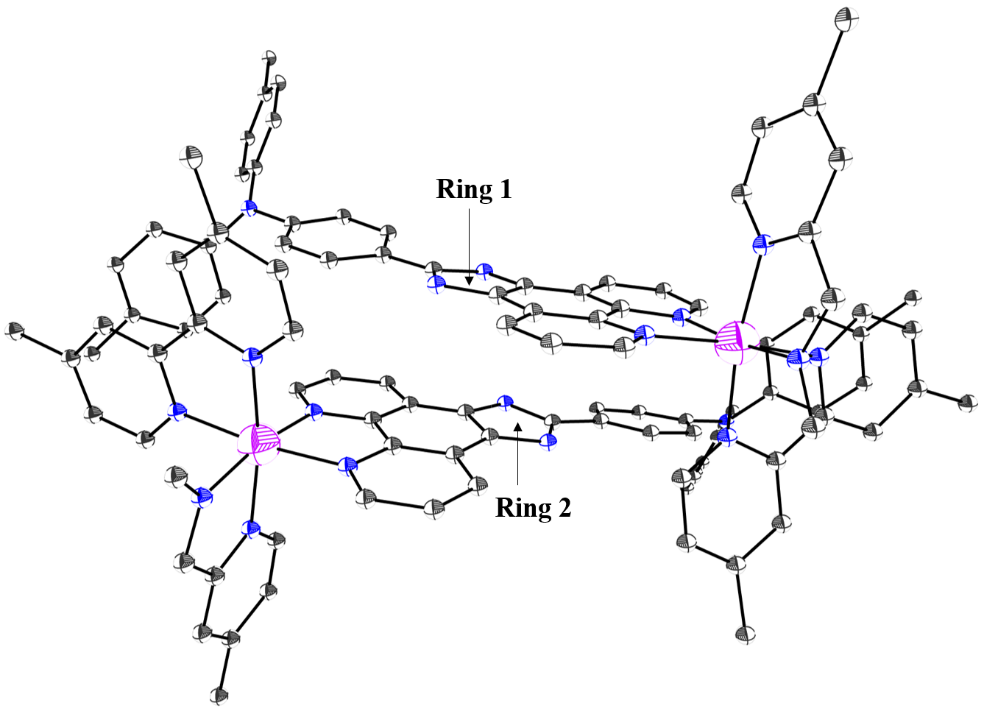


**FIGURE. S3.** Crystal structure diagram.


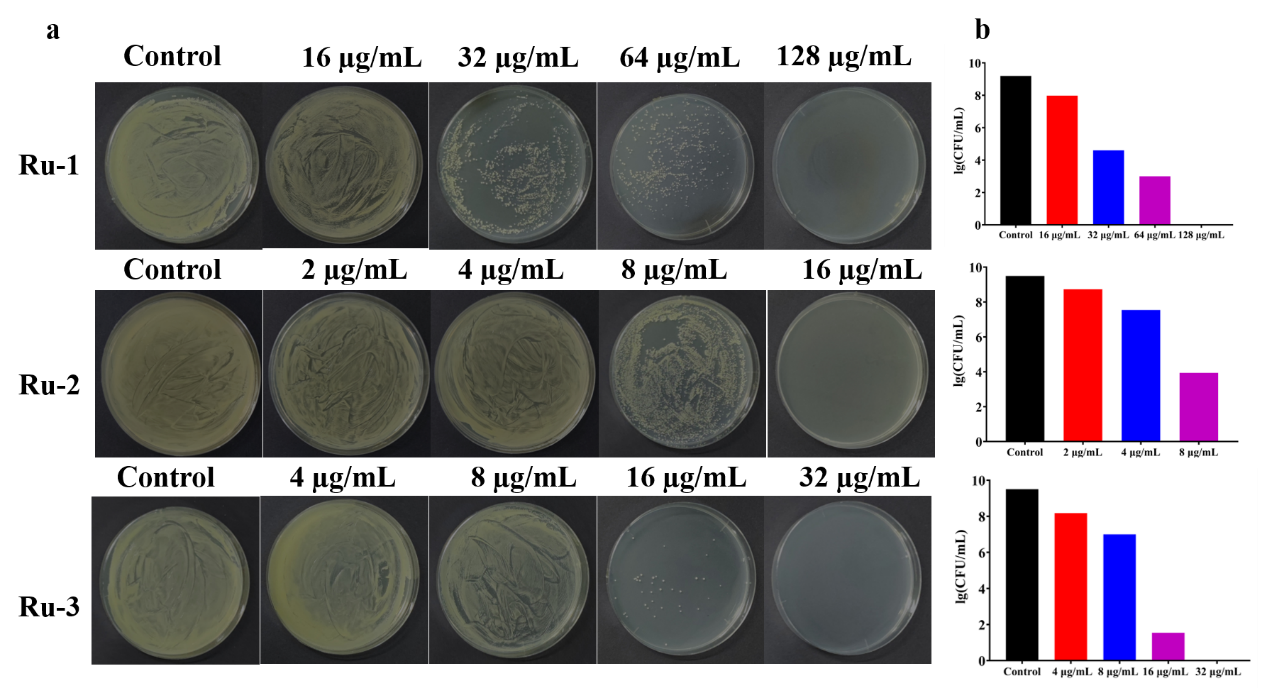


**FIGURE. S4.** Killing of *S. aureus* strain by ruthenium complexes.


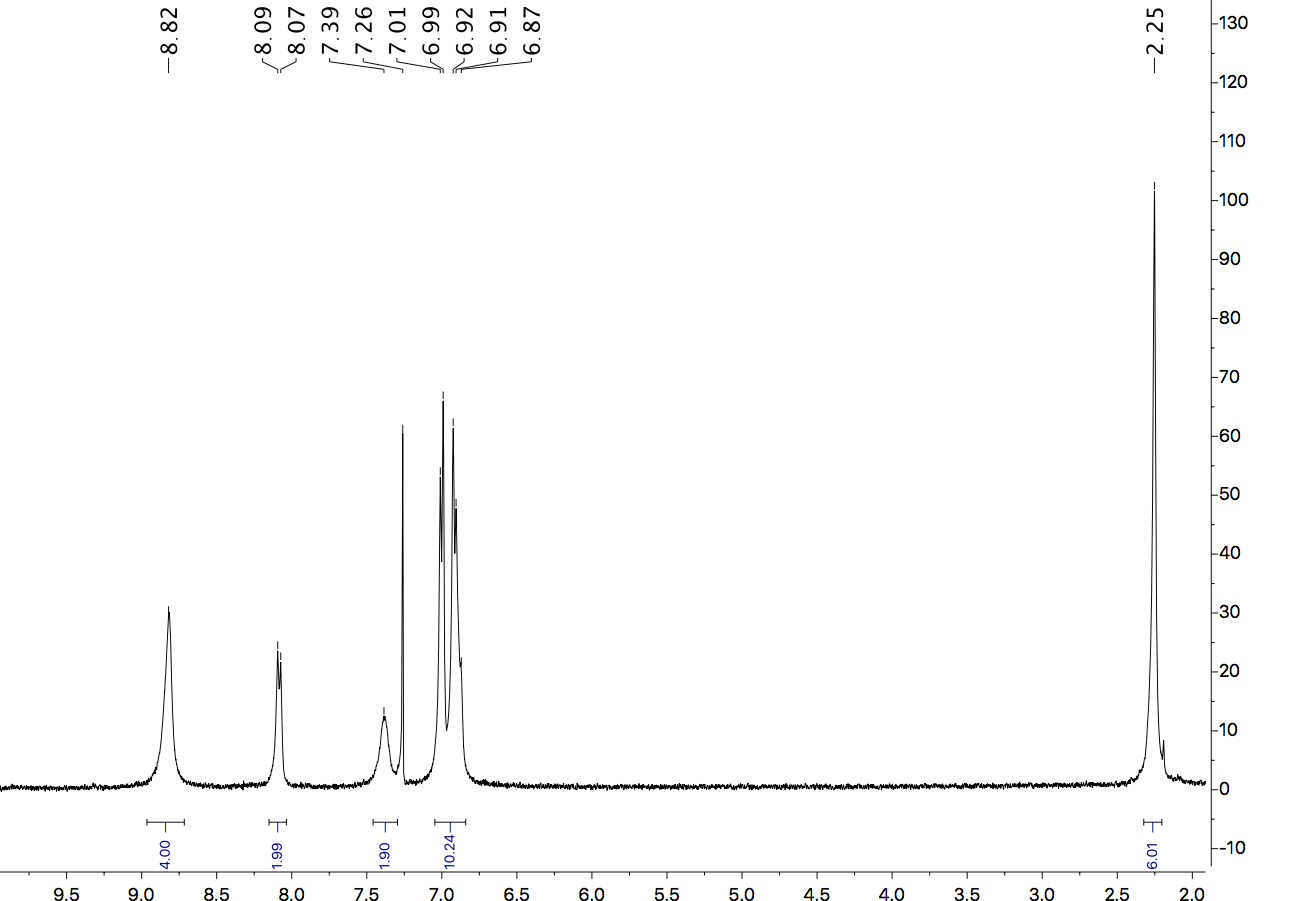


**FIGURE. S5.** ^1^H NMR spectrum of PMA.


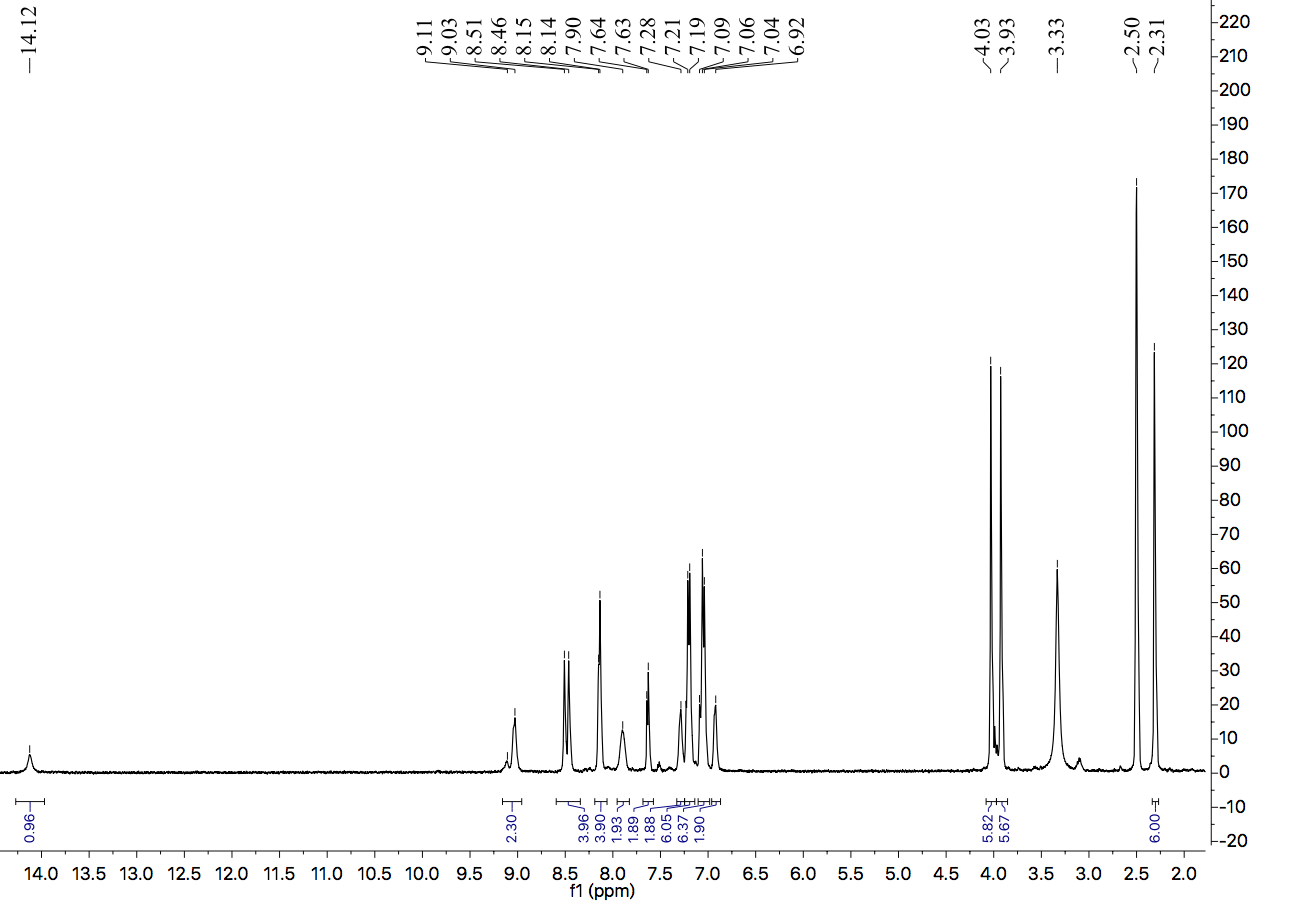

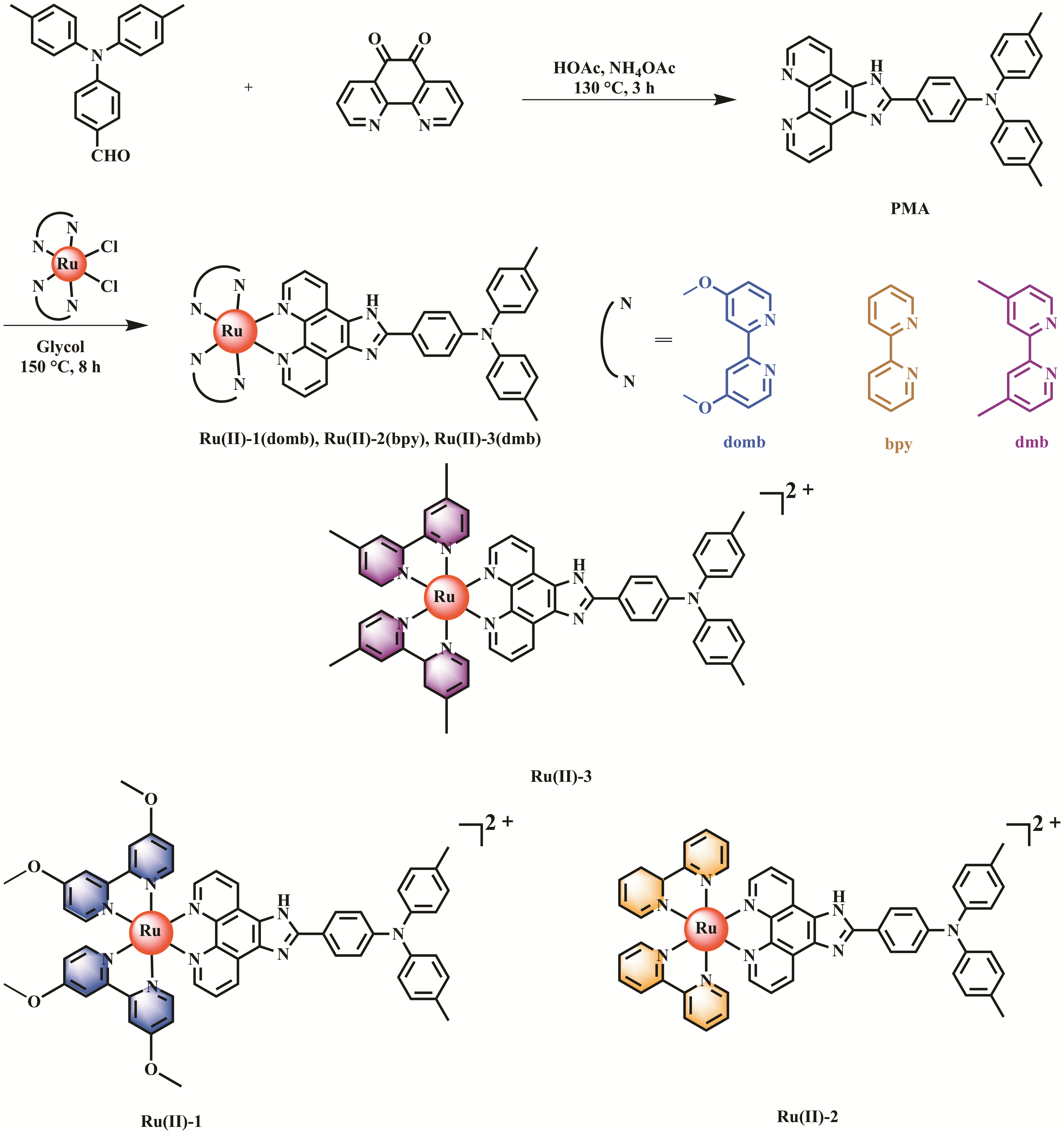


**FIGURE. S6.** ^1^H NMR spectrum of **Ru-1**.


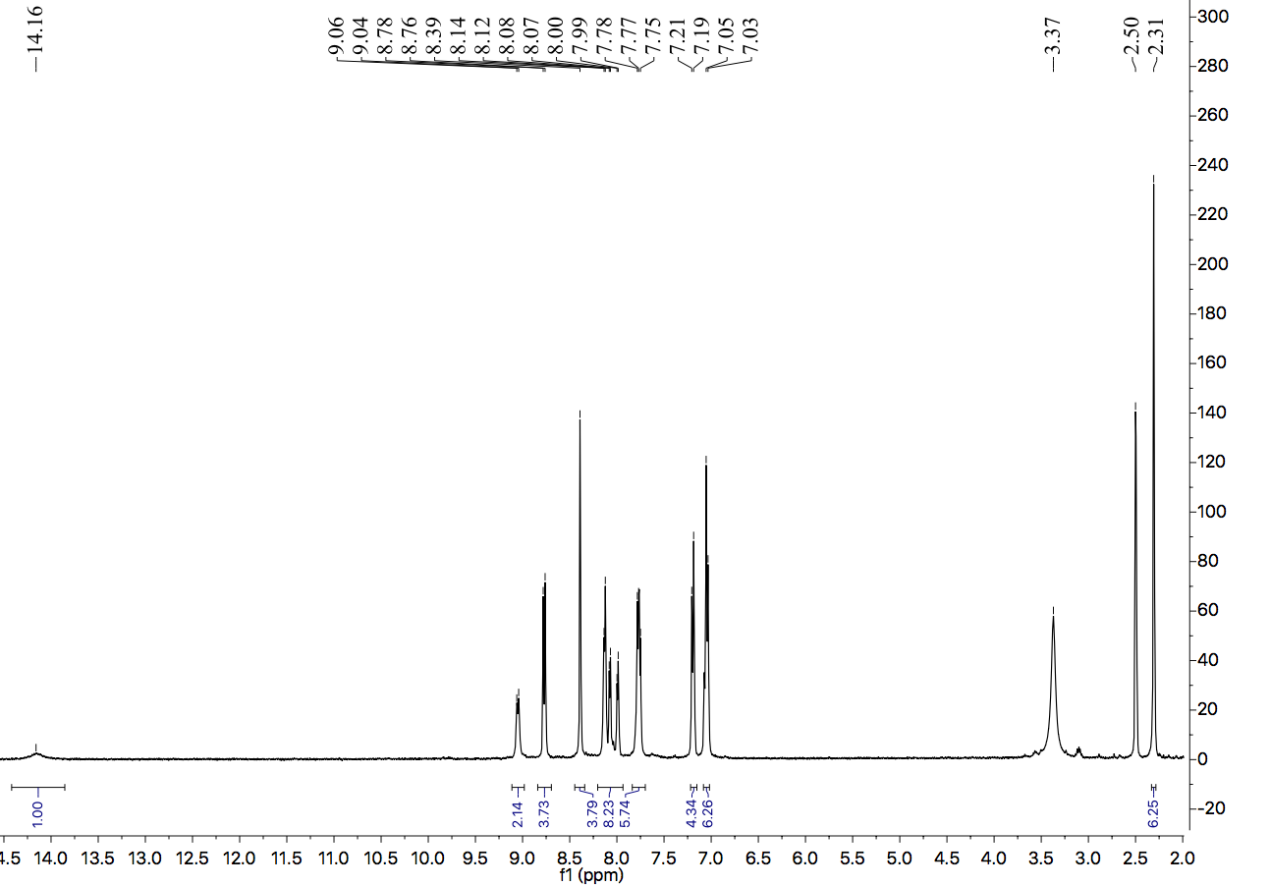

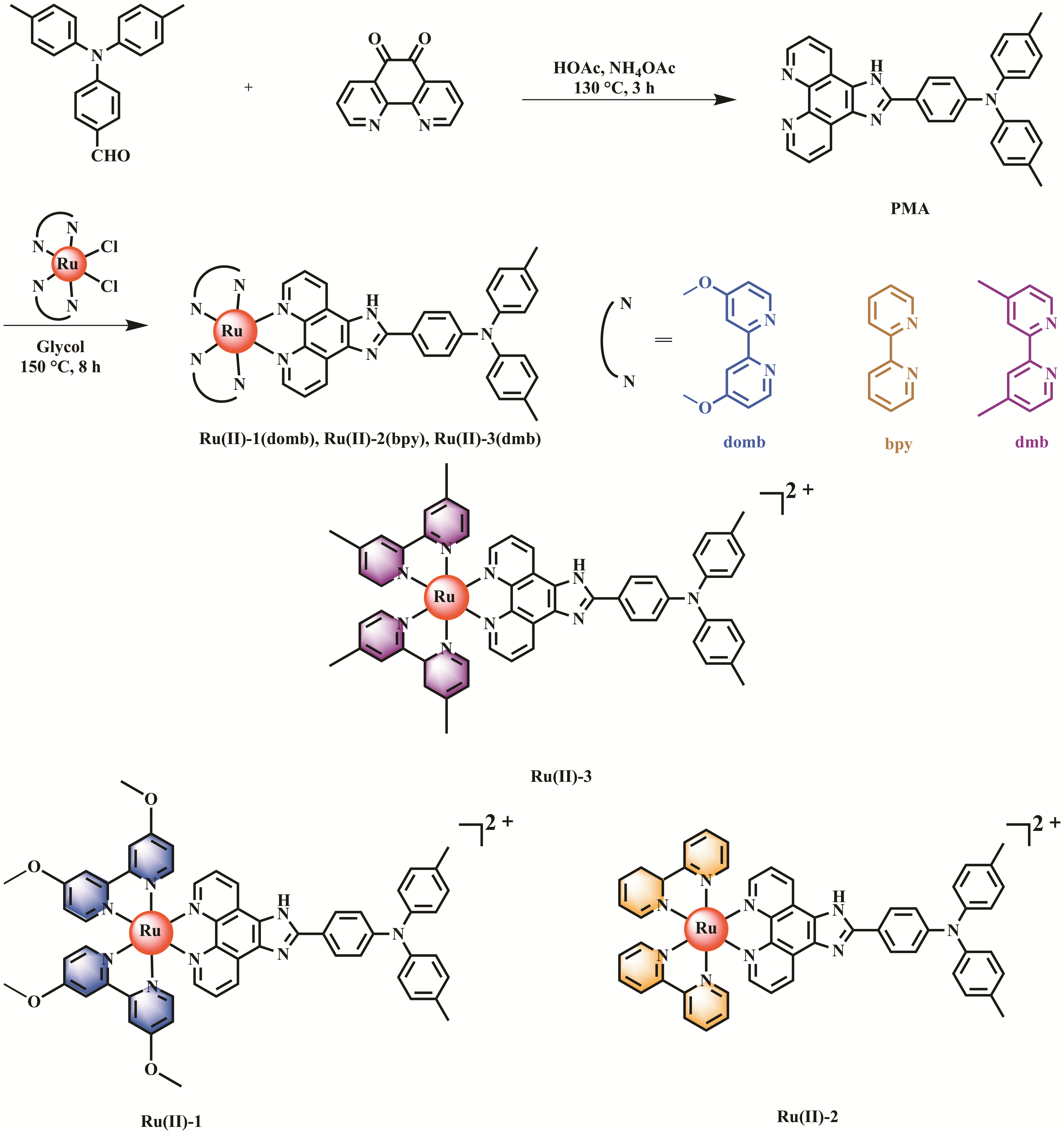


**FIGURE. S7.** ^1^H **NMR** spectrum of **Ru-2**.


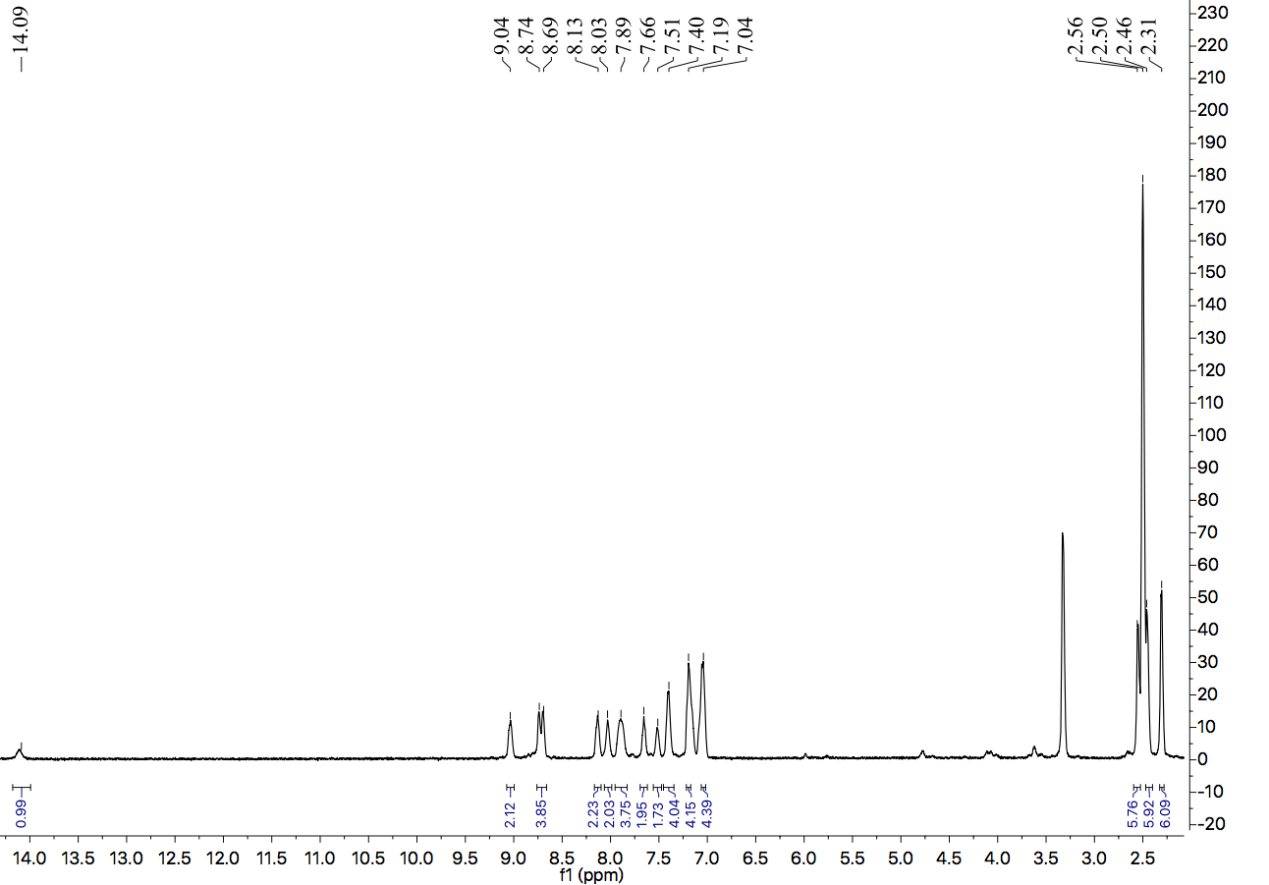

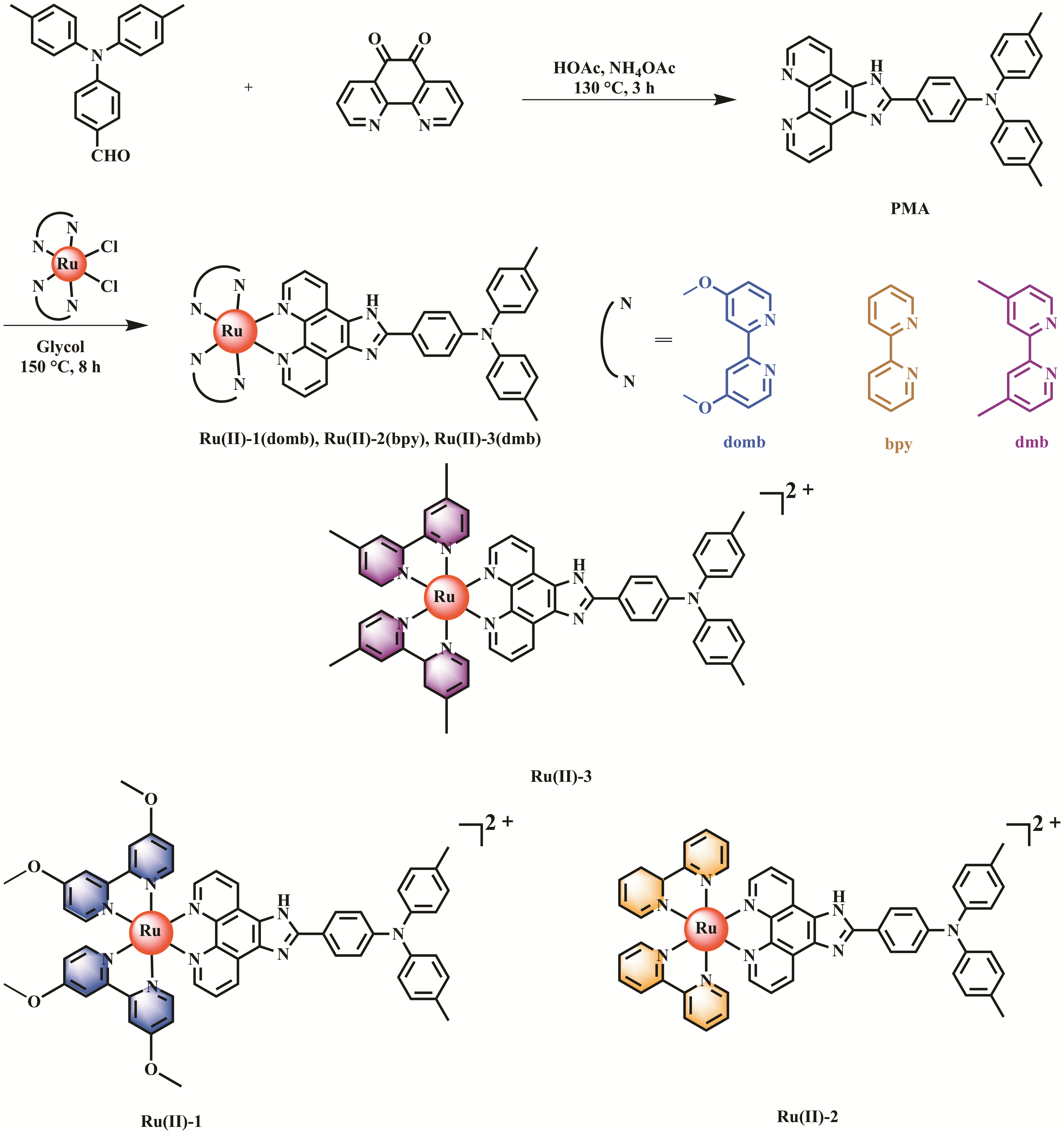


**FIGURE. S8.** ^1^H **NMR** spectrum of **Ru-3**.


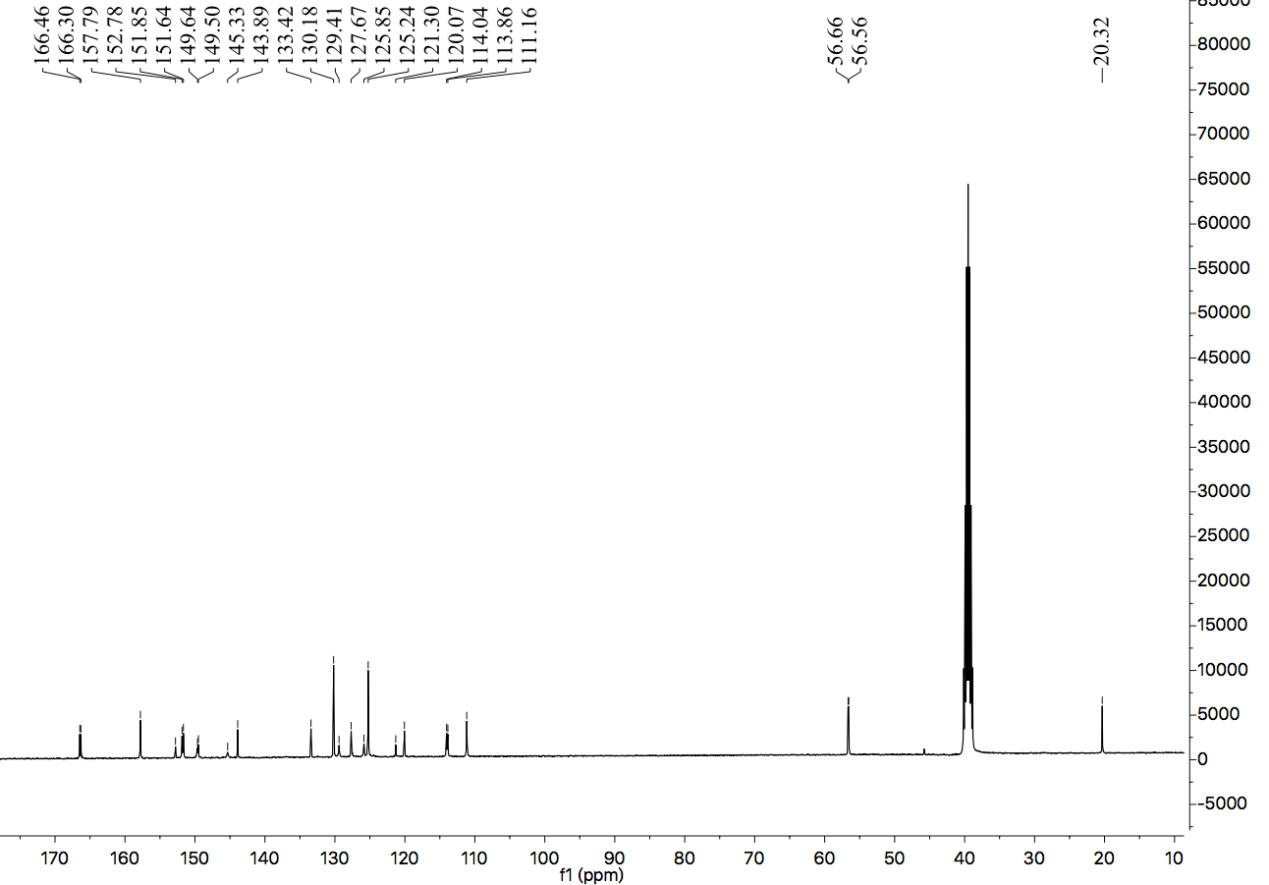


**FIGURE. S9.** ^13^C NMR spectrum of spectrum of **Ru-1.**


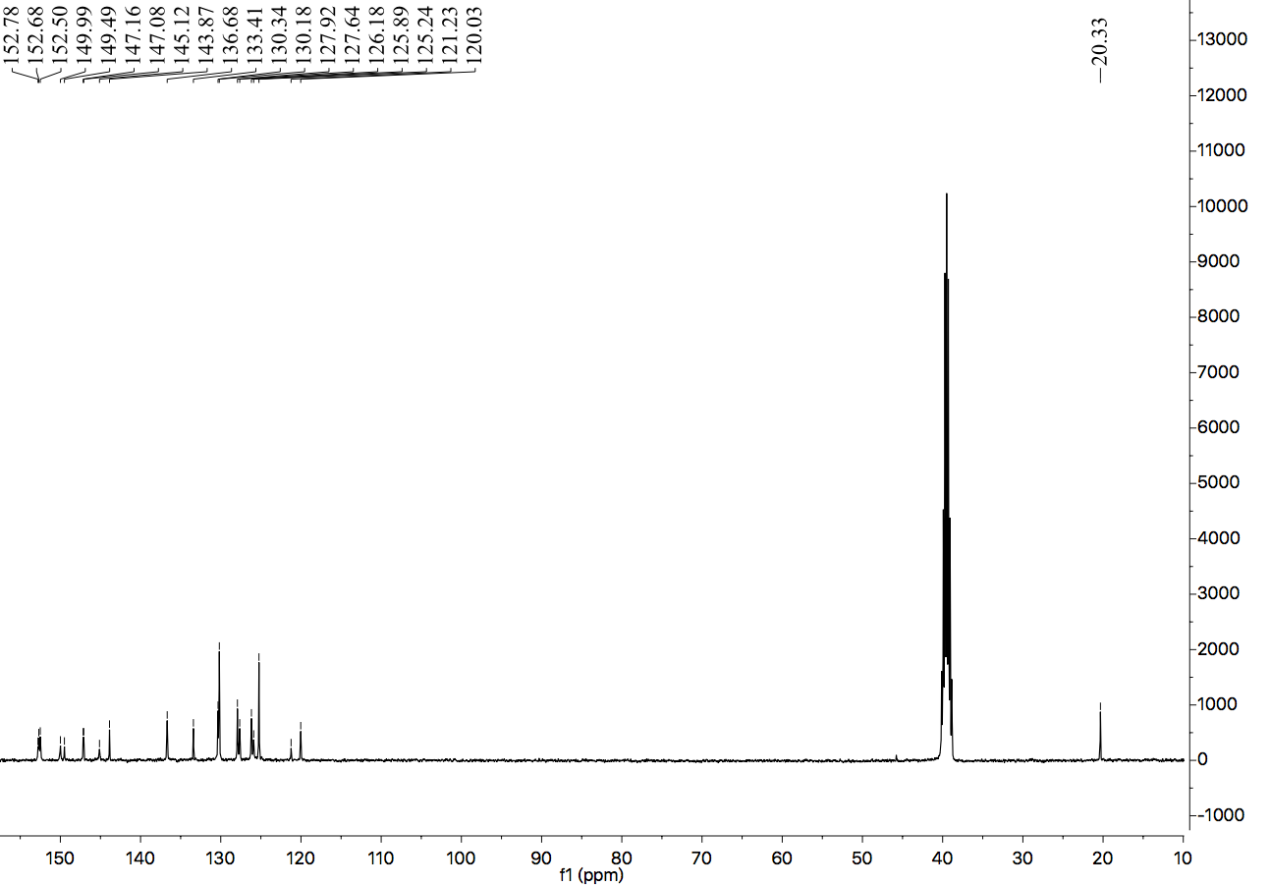

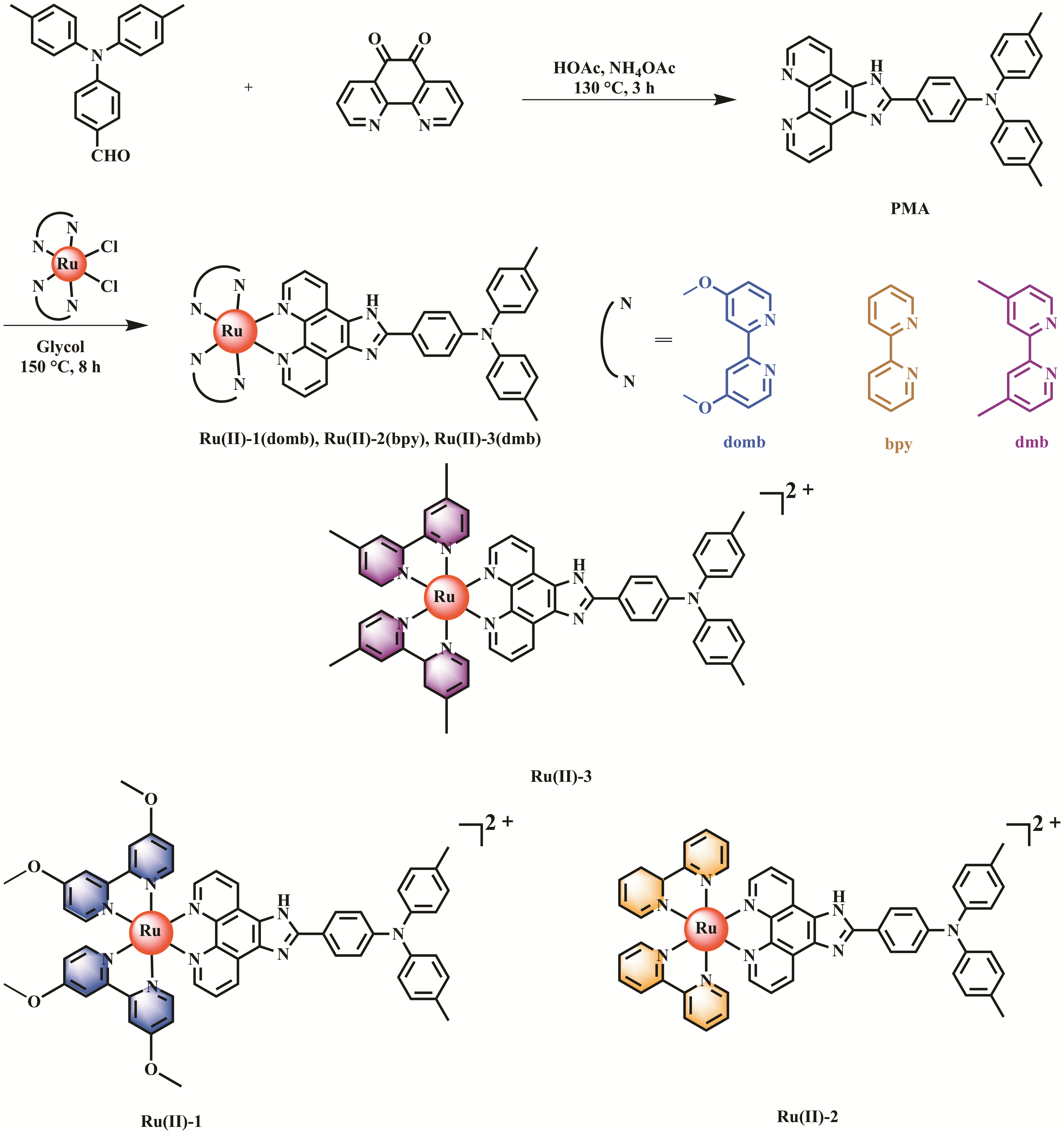


**FIGURE. S10.** ^13^C NMR spectrum of spectrum of **Ru-2**.


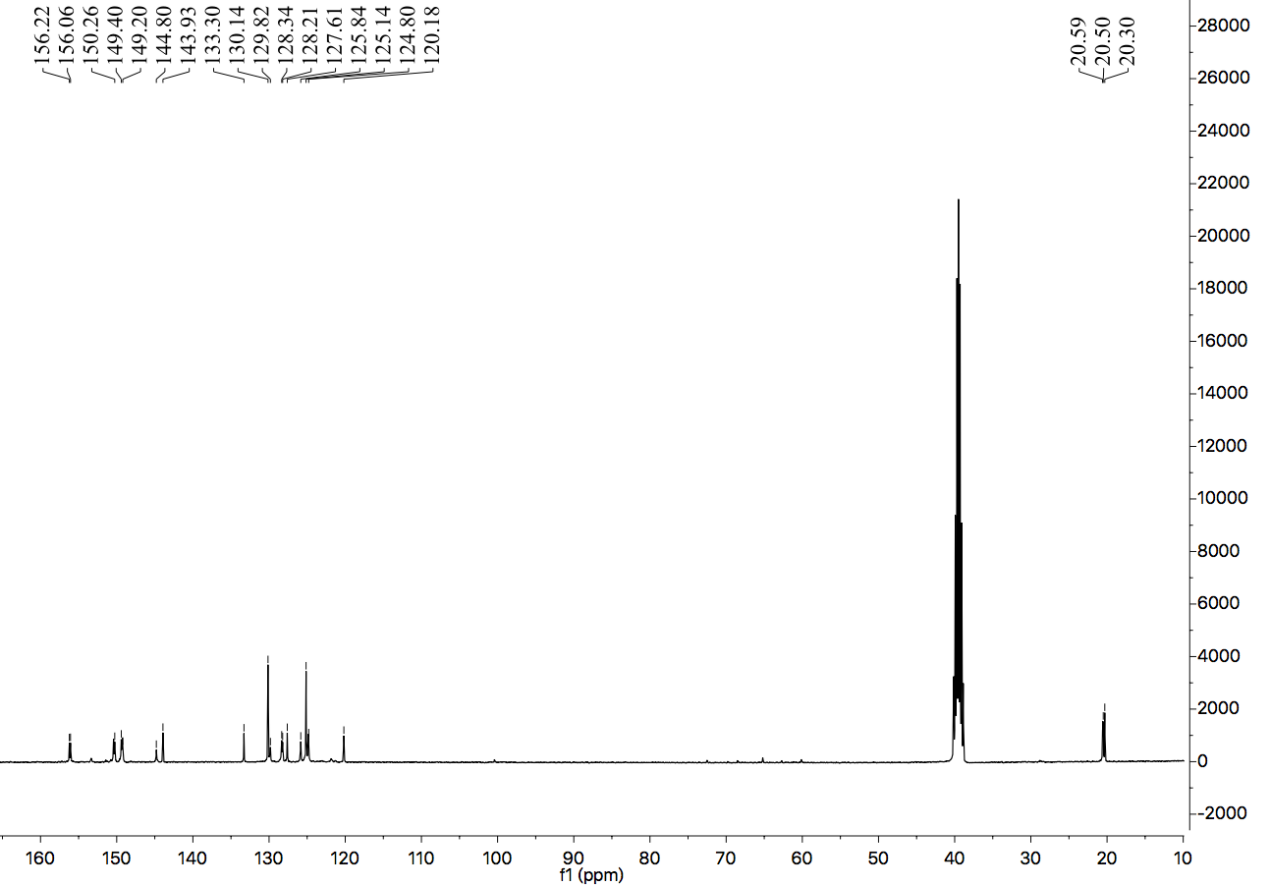

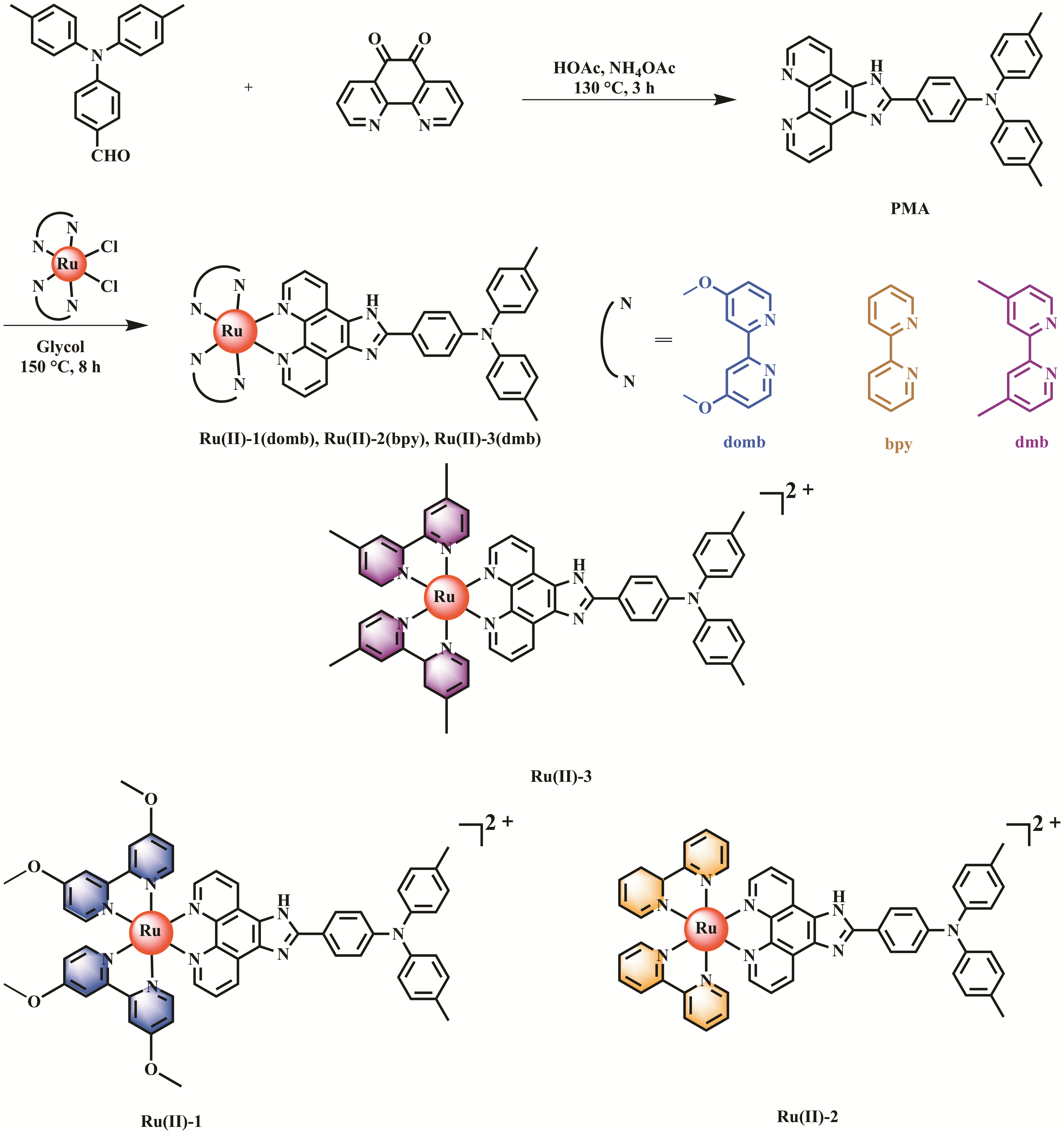


**FIGURE. S11.** ^13^C NMR spectrum of spectrum of **Ru-3**.


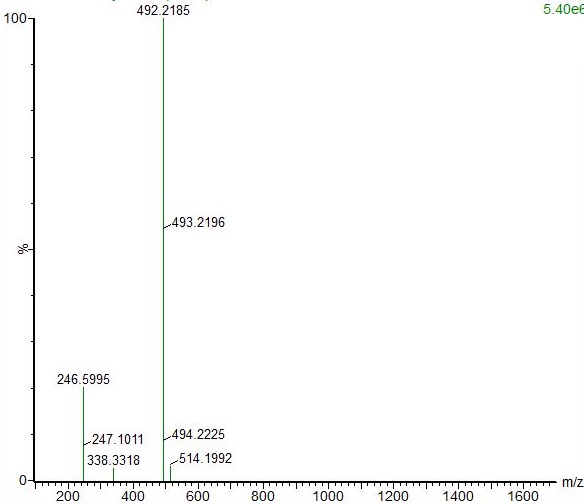

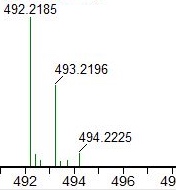

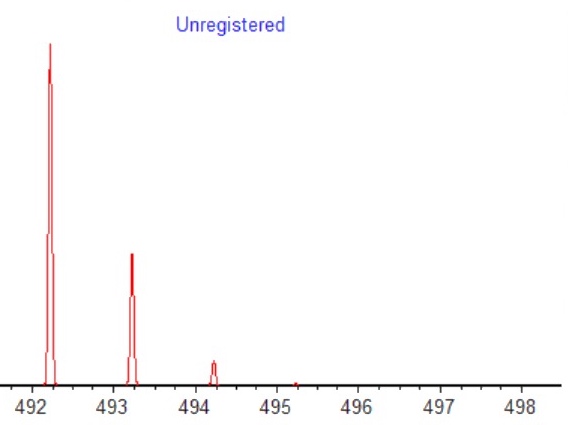


**FIGURE. S12.** HRMS spectrum of PMA.


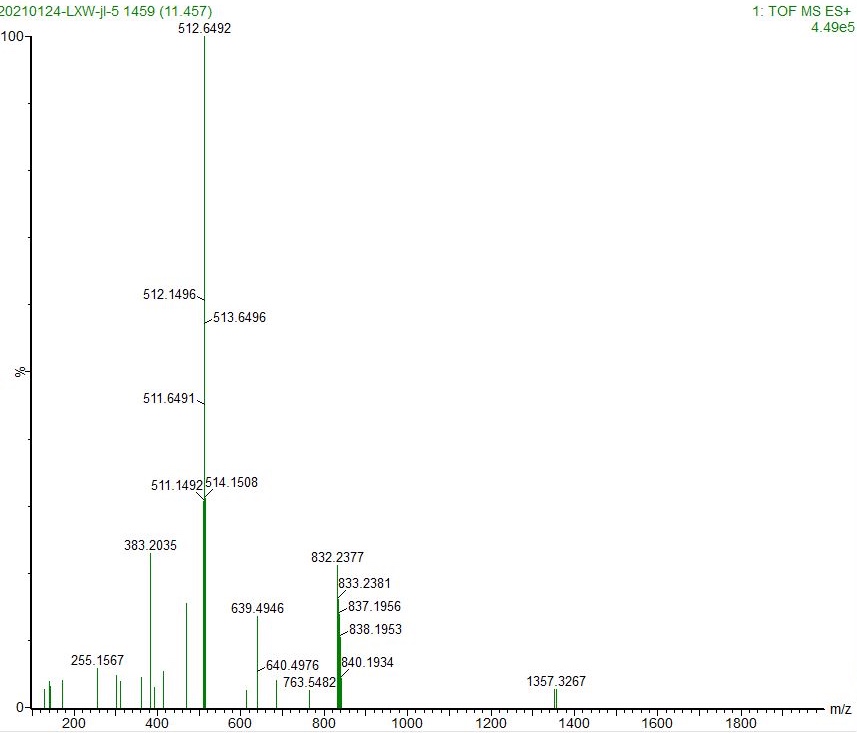

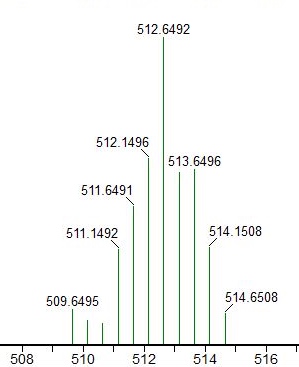

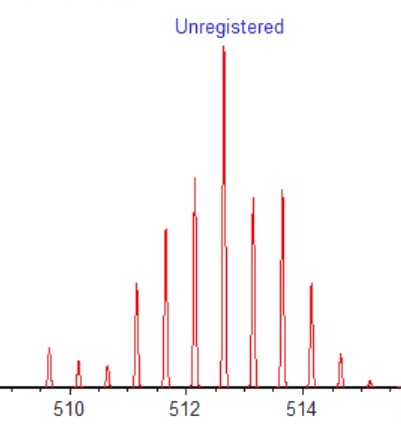


**FIGURE. S13.** HRMS spectrum of **Ru-1**.


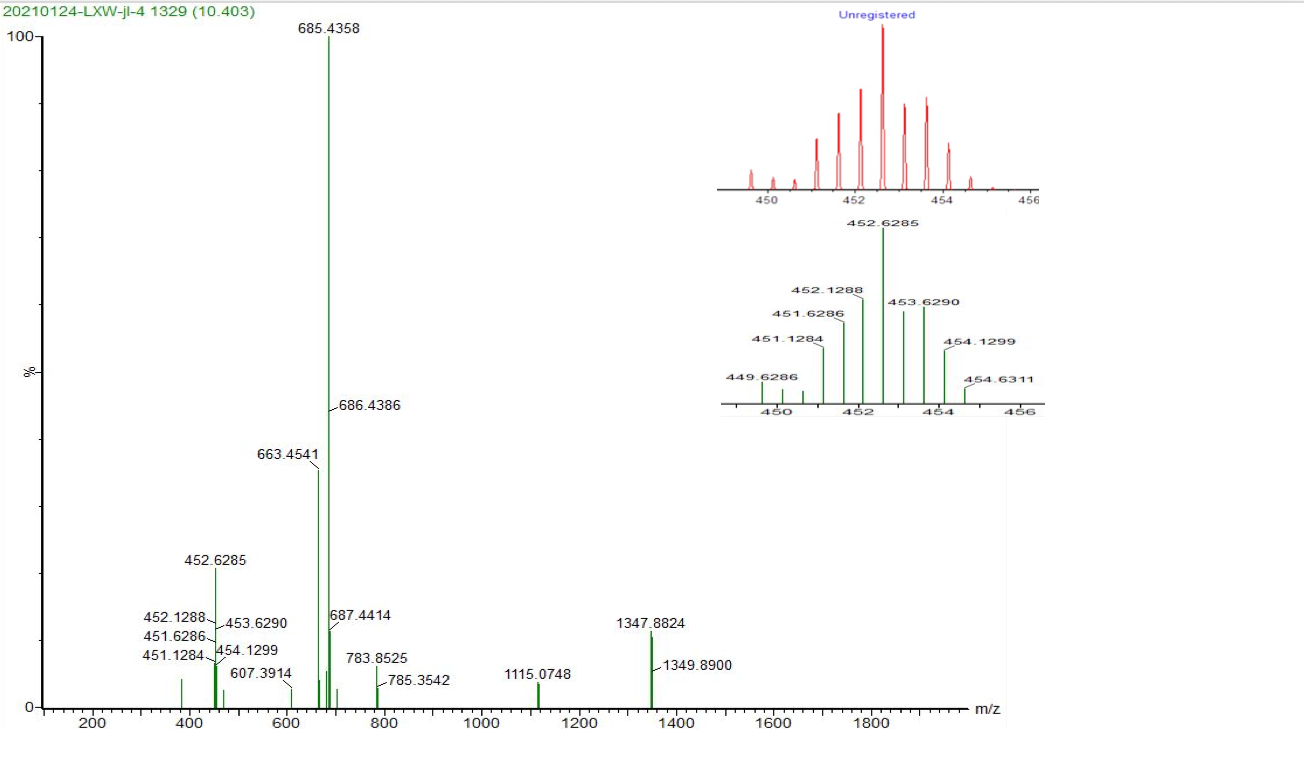


**FIGURE. S14.** HRMS spectrum of **Ru-2**.


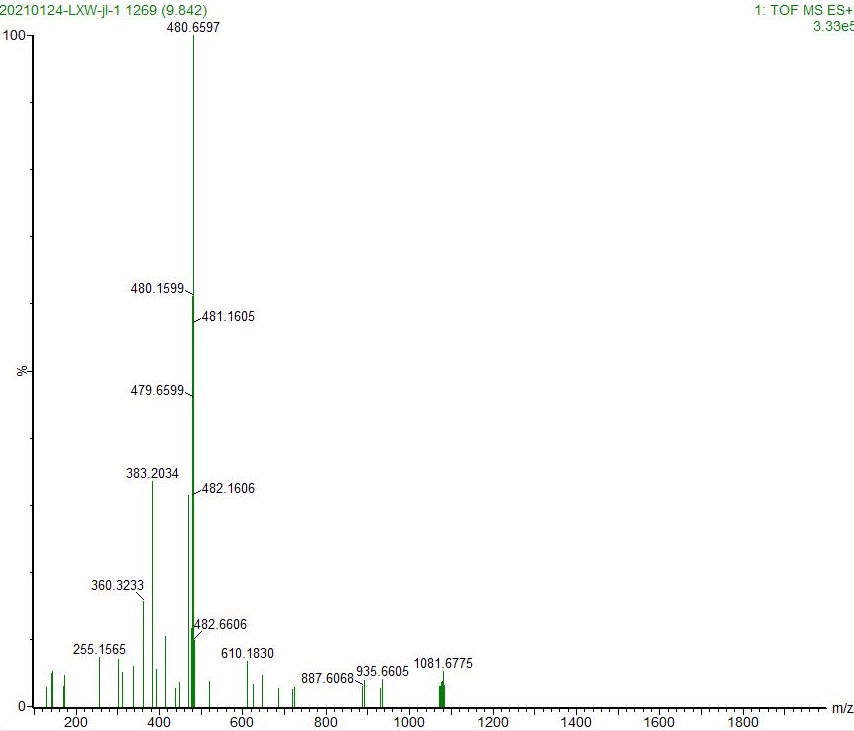

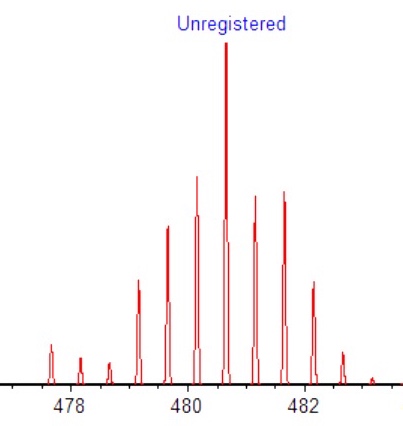

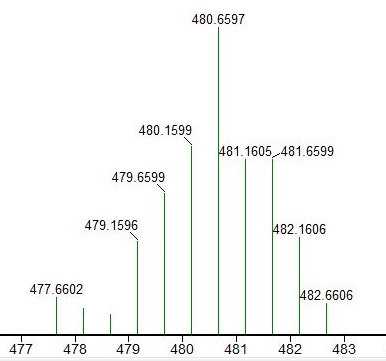


**FIGURE. S15.** HRMS spectrum of **Ru-3**.


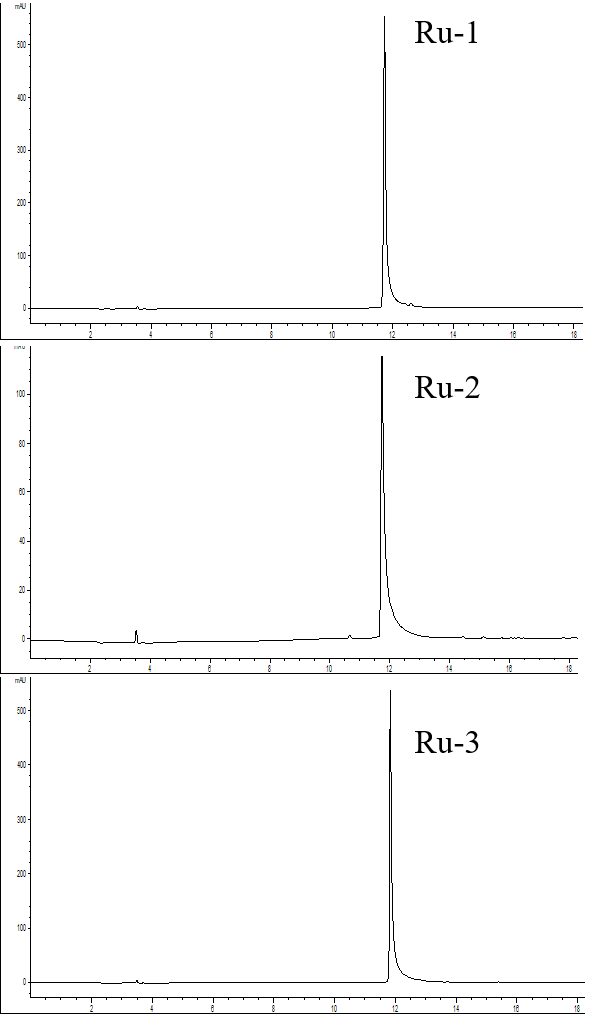


**FIGURE. S16.** HPLC spectrum of three compounds.


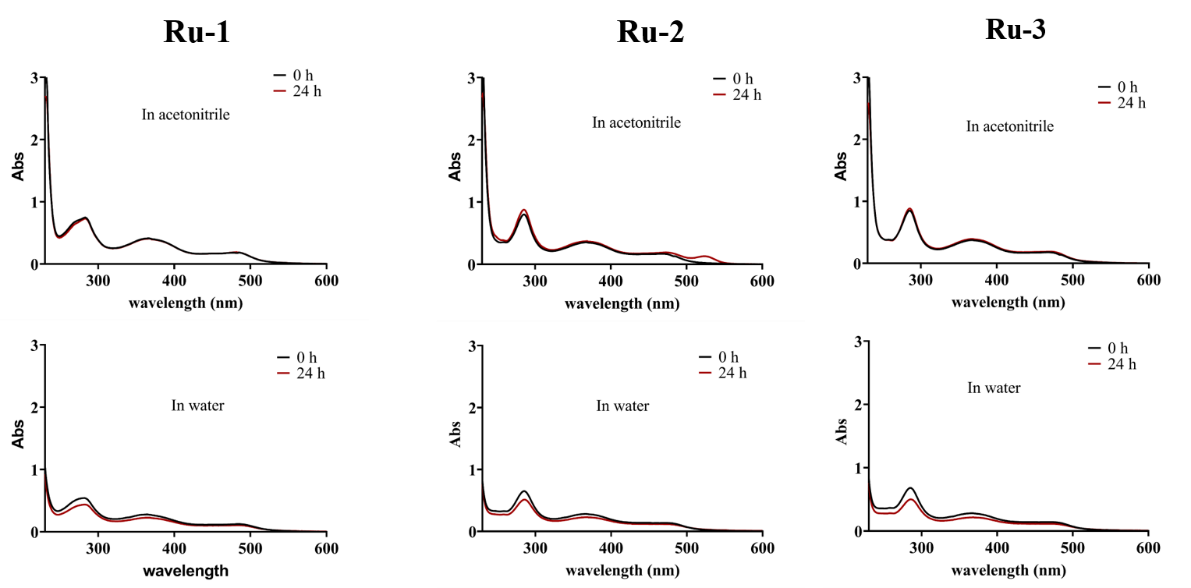


**FIGURE. S17.** Stability of three compounds.

**Table S1. Crystallographic data of Ru-3.**

| Empirical formula | C_57_H_49_F_12_N_9_P_2_Ru |
| --- | --- |
| Formula weight | 1251.06 |
| Crystal system | Monoclinic |
| Space group | C2 |
| a/Å b/Å c/Å | 49.386(2), 17.9476(9), 15.6461(7) |
| α/(°) β/(°) γ/(°) | 90, 99.2970(10), 90 |
| Volume /Å^3^ | 13685.9(11) |
| Z | 8 |
| μ/mm^-1^ | 0.347 |
| F(000) | 5088.0 |
| Radiation | MoKα (λ = 0.71073) |
| Index ranges | -57 ≤ h ≤ 58, -21 ≤ k ≤ 18, -18 ≤ l ≤ 18 |
| Goodness-of-fit on F^2^ | 0.986 |
| Independent reflections | 22079 [R_int_ = 0.0790, R_sigma_ = 0.1222] |
| Final R indexes [I>=2σ (I)] | R_1_ = 0.0699, wR_2_ = 0.1606 |
| Final R indexes [all data] | R_1_ = 0.1339, wR_2_ = 0.2021 |
| Data/restraints/parameters | 22079/588/1660 |
| Largest diff. peak/hole / e Å^-3^ | 0.48/-0.42 |

**Table S2. Selected bond lengths [Å] Ru-3 and Selected angles (°) for Ru-3.**

| Bond | Angle/(˚) | Bond | Angle/(˚) | Bond | Length/nm |
| --- | --- | --- | --- | --- | --- |
| N1-Ru1-N13 | 173.0(4) | N13-Ru1-N12 | 95.3(4) | Ru1-N4 | 2.032(11) |
| N1-Ru1-N13 | 173.0(4) | N13-Ru1-N12 | 95.3(4) | Ru1-N4 | 2.032(11) |
| N1-Ru1-N18 | 94.1(4) | N18-Ru1-N12 | 78.3(5) | Ru1-N10 | 2.031(9) |
| N4-Ru1-N1 | 79.8(4) | N18-Ru1-N13 | 90.8(4) | Ru1-N12 | 2.066(8) |
| N4-Ru1-N12 | 94.2(5) | N8-C58-C78 | 123.8(12) | Ru1-N13 | 2.063(10) |
| N4-Ru1-N13 | 96.0(4) | N8-C58-N9 | 114.4(10) | Ru1-N18 | 2.042(10) |
| N4-Ru1-N18 | 170.4(4) | N9-C58-C78 | 121.8(13) | N17-C35 | 1.43(2) |
| N10-Ru1-N1 | 95.4(4) | C35-N17-C89 | 118.1(14) | N17-C83 | 1.40(2) |
| N10-Ru1-N4 | 93.8(4) | C83-N17-C35 | 120.3(13) | N17-C89 | 1.438(16) |
| N10-Ru1-N12 | 170.8(4) | C83-N17-C89 | 120.3(13) | C58-C78 | 1.453(17) |
| N10-Ru1-N13 | 79.3(4) | N9-C58-C78 | 121.8(13) |  |  |
